# A transcription factor atlas of stem cell fate in planarians

**DOI:** 10.1016/j.celrep.2024.113843

**Published:** 2024-02-23

**Authors:** Hunter O. King, Kwadwo E. Owusu-Boaitey, Christopher T. Fincher, Peter W. Reddien

**Affiliations:** 1 Howard Hughes Medical Institute, Chevy Chase, MD, USA; 2 Whitehead Institute for Biomedical Research, Cambridge, MA, USA; 3 Department of Biology, Massachusetts Institute of Technology, Cambridge, MA, USA; 4 Department of Brain and Cognitive Sciences, Massachusetts Institute of Technology, Cambridge, MA, USA; 5 Harvard/MIT MD-PhD Program, Harvard Medical School, Boston, MA, USA; 6 These authors contributed equally; 7 Lead contact

## Abstract

Whole-body regeneration requires the ability to produce the full repertoire of adult cell types. The planarian *Schmidtea mediterranea* contains over 125 cell types, which can be regenerated from a stem cell population called neoblasts. Neoblast fate choice can be regulated by the expression of fate-specific transcription factors (FSTFs). How fate choices are made and distributed across neoblasts versus their post-mitotic progeny remains unclear. We used single-cell RNA sequencing to systematically map fate choices made in S/G_2_/M neoblasts and, separately, in their post-mitotic progeny that serve as progenitors for all adult cell types. We defined transcription factor expression signatures associated with all detected fates, identifying numerous new progenitor classes and FSTFs that regulate them. Our work generates an atlas of stem cell fates with associated transcription factor signatures for most cell types in a complete adult organism.

## In brief

Planarian stem cells generate 125+ differentiated cell types comprising the entire body. The stem cells produce postmitotic progenitors that migrate and differentiate. King et al. systematically identified fate choices within stem cells and post-mitotic progenitors and identified transcription factor expression signatures associated with fate choices for the complete animal.

## Graphical Abstract

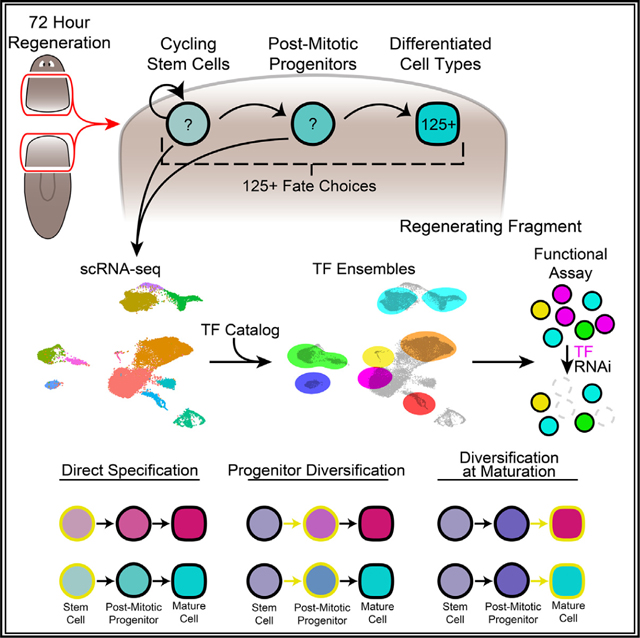

## INTRODUCTION

Adult stem cells in many regenerative organisms must be capable of making a large array of possible fate choices. Planarians take this challenge to the extreme, where stem cells and/or their post-mitotic descendant cells must choose between over 125 possible fates.^1^ Planarians can regenerate any missing body part, including the entire animal from small body fragments and also constitutively replace all tissues through homeostatic tissue turnover. Adult stem cells called neoblasts are responsible for the production of all adult somatic cell types in both regeneration and tissue turnover.^2,3^

In many developing animals, the enormous challenge of generating cellular diversity is accomplished gradually and can involve progressive fate restriction, sometimes with a long lineage hierarchy.^4^ In regeneration, by contrast, new tissues can return quickly and concurrently.^5,6^ For instance, in planarians, new cell types can differentiate within 3 days despite neoblast cell division taking 12–24 hours.^5,7,8^ Furthermore, the identity of missing cell types at wounds is unpredictable, potentially making a strict hierarchical lineage impractical for tailoring production to the identity of missing cell types. Therefore, addressing the mechanisms of fate choice specification in stem and progenitor cells is central for understanding regeneration.

Substantial data indicate that neoblasts are a major site of fate specification in planarians.^5^ Neoblasts are molecularly and functionally heterogeneous, being composed of distinct subsets called specialized neoblasts. Specialized neoblasts express transcription factors (TFs) associated with particular fates, termed fate-specific transcription factors (FSTFs).^9^ Many FSTFs are required for the formation of different cell types.^1,10–31^ Specialized neoblasts, therefore, serve as the precursors for mature cell types. Not all FSTFs have been shown to have a functional role in specification; many have been shown to have restricted expression that spans multiple cell types, but are still informative in defining the transcriptional signature characteristic of different specialized neoblasts.

FSTF expression is common in S/G_2_/M neoblasts and neoblast specification occurs as cells transit through the cell cycle.^9^ Investigation into fate choice in neoblasts includes hierarchical models for neoblast potency.^32^ By contrast, experiments studying FSTF activation, specialized neoblast potential, and asymmetric divisions support a non-hierarchical model called the single-step fate model, in which neoblasts can specify a particular fate during the cell cycle and divide asymmetrically to retain potency.^9^ Neoblast division can also result in post-mitotic non-neoblast progeny cells that rapidly cease expression of neoblast-specific genes.^9^ These non-dividing cells are progenitors that can migrate and differentiate according to their fate.^5^ Post-mitotic progenitor cells can be isolated with fluorescence-activated cell sorting (FACS), but whether fate specification occurs in these cells remains little explored.

Comprehensive adult planarian single-cell RNA sequencing (scRNA-seq) estimated that ~125 distinct mature cell types/states exist.^1^ scRNA-seq has also made it possible to identify neoblast subtypes for the major tissue classes of the animal.^1,32–34^ However, little additional structure has been described for such neoblast clusters, leaving unclear the scope of specification that happens in neoblasts. For instance, are all ~125 possible fate choices made in the neoblasts? Alternatively, are some choices made in post-mitotic progenitors? What TF expression signatures and dynamics characterize and regulate the generation of cell-type diversity as progenitors mature and potentially diversify over time? To address these questions, we performed scRNA-seq on dividing neoblasts (S/G_2_/M) and G_0_ post-mitotic neoblast progeny during regeneration and identified novel cell states and TF expression signatures in these stages. We find differences across several major tissue types in how cell-type diversity is generated. We assembled a curated catalog of all putative planarian TFs and utilized it in conjunction with scRNA-seq data to uncover new FSTFs and specialized neoblast populations. Our results generate a stem cell-fate atlas for the majority of cell types of a complete adult animal.

## RESULTS

### scRNA-seq of S/G_2_/M neoblasts identifies diverse specialized neoblast classes

**Figure 1.**
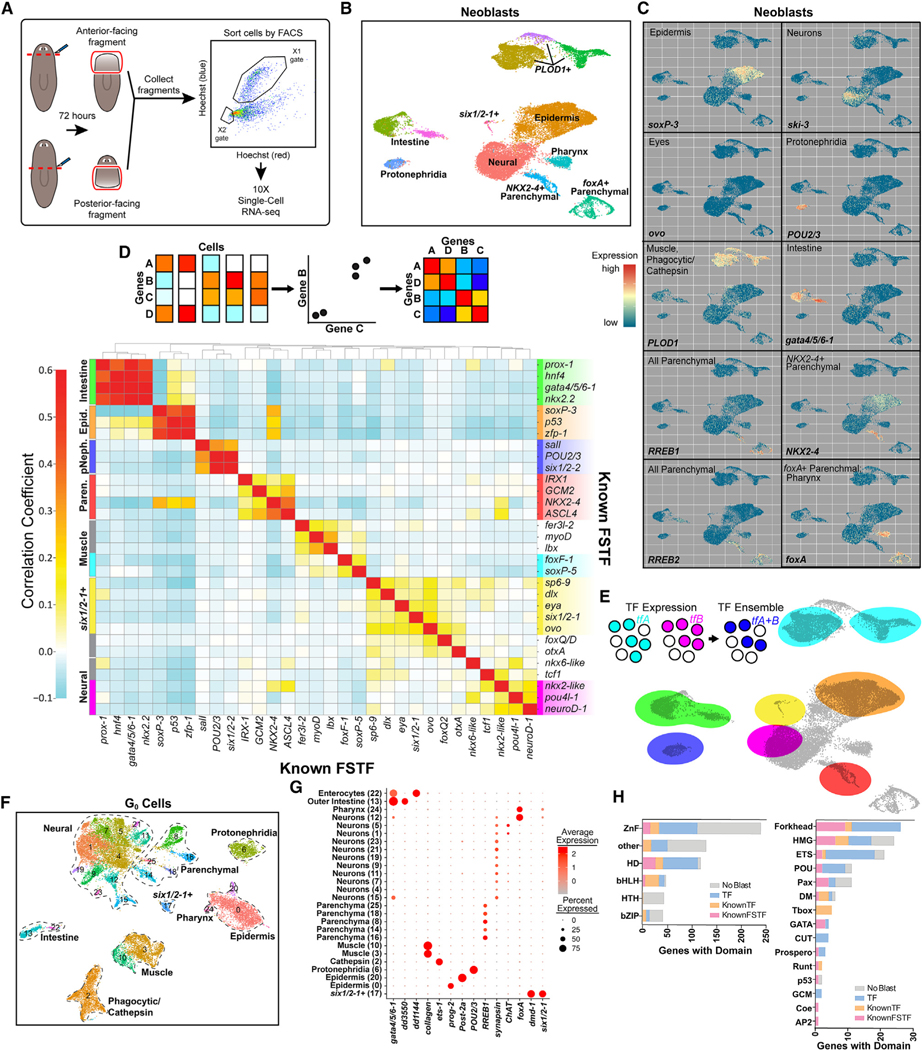
scRNA-seq of S/G_2_/M neoblasts and G_0_ progeny (A) Schematic of experimental workflow. (B) UMAP plot of X1 neoblasts. (C) Expression (red, high; blue, low) of marker genes. (D) Top: schematic of gene correlation analysis procedure. Bottom: correlation analysis heatmap of subset of known FSTFs across S/G_2_/M cells. (E) Top: illustration defining TF ensemble as coexpression of multiple TFs. Bottom: TF ensemble expression domain overlaid onto UMAP plot. Ensemble colors correspond to ensembles of same color in (D). (F) UMAP plot of G_0_ cells. (G) Dot plot of known tissue marker expression across G_0_ clusters. (H) Categorization of genes within TF catalog by family and previous identification (known TF or FSTF) or BLAST similarity to human TF.

Recent work showed that FSTF expression is high in S/G_2_/M neoblasts, suggesting that the complete set of neoblast fate choices could be investigated in the 4C neoblast stage.^9^ S/G_2_/M neoblasts can be isolated with Hoechst labeling and FACS based on their 4C DNA content (the X1 gate) (Figures 1A, S1A, and S1B).^35^ Given these attributes, we selected 4C neoblasts to characterize fate choices made in planarian stem cells. We purified neoblasts 72 hours after anterior or posterior amputation, yielding neoblasts involved in anterior and posterior regeneration, respectively. This time point involves substantial neoblast amplification and reestablishment of positional information that influences regeneration outcomes.^36^ We analyzed 23,873 S/G_2_/M neoblasts that formed 12 distinct clusters (Figure 1B).

Planarian tissues are categorized into nine major classes: neoblasts, epidermis, muscle, neurons, intestine, protonephridia (a filtration organ system), phagocytic/*cathepsin*+ cells, glandular/parenchymal cells, and pharynx.^1,33^ Within each major class, there exist numerous distinct cell types. We clustered single-cell neoblast transcriptomes and found that neoblasts for all eight major differentiated planarian tissue types were represented by at least one large cluster (Figures 1B, 1C, and S1C; Table S1). Specialized neoblasts for rare cell types, such as photoreceptor neurons, were present in the S/G_2_/M neoblast transcriptomes as well, indicating that even the rarest specialized neoblast classes should be present in this dataset. There was no cluster identified that lacked cells with an expression signature indicative of specialization toward a major tissue class (Figures 1B, 1C, and S1C).

Because multiple FSTFs often characterize a cell type, we hypothesized that ensembles of correlated TFs could be used to identify new cell types or states, as well as to identify FSTFs associated with them. We computed pairwise correlations between previously identified FSTFs across neoblasts and performed hierarchical clustering to group correlated/coexpressed FSTFs into modules (Figure 1D). FSTFs associated with fate specification of the same tissue displayed correlated expression and clustered together into modules representing the different tissues that they mutually specify. We then identified cells coexpressing components of the TF ensembles to annotate regions of cells in Uniform Manifold Approximation and Projection (UMAP) visualizations (Figure 1E). This approach identified UMAP regions containing specialized neoblasts for the intestine, epidermis, protonephridia, parenchyma, muscle, eyes, and neurons. By identifying cells through the shared expression of a module of TFs instead of individual canonical TFs, cells could more precisely be classified to a specific cell type, because many individual TFs were expressed in multiple cell types. For example, epidermal-specialized neoblasts express *zfp-1*, *p53*, and *soxP-3*, but cells expressing each individual gene were not all epidermis fated (70%, 67%, and 86% were epidermis fated, respectively; Figures S1D–S1F). However, 97% of cells expressing all three FSTFs were epidermis fated. This approach was applied broadly below in ascribing cell identity and for identifying FSTFs associated with neoblast states.

### Defining the transcriptomes of post-mitotic progenitors

4C neoblasts produce post-mitotic (post-neoblast) G_0_ cells that are progenitors for differentiated cell types. Whether all cell-fate decisions are made in the neoblasts or whether fate decisions continue to be made in G_0_ progenitor stages is unknown. Investigating these G_0_ cells is therefore critical for understanding how the complete complement of cell-fate choices emerge. We used FACS to isolate 31,084 G_1_/G_0_ cells (the X2 gate)^35^ during anterior and posterior regeneration. G_0_ cells were computationally isolated from G_1_ cells as previously described,^9^ and clustering of G_0_ cells resulted in 26 cell clusters (Figure 1F), which represented progenitors for the known tissue types of the animal (Figure 1G). FSTFs and differentiated cell-type-specific markers were often coexpressed in G_0_ clusters (Figure S1G). Progenitors for rare cell types (e.g., photoreceptors) were present, suggesting that this dataset contains even the rarest progenitor classes.

### *In silico* identification of planarian transcription factors

Because TF expression can distinguish classes of progenitors, we generated an *in silico* planarian TF catalog (Figure S1H). We utilized this gene set to identify neoblast types and the TFs that define them. Additional planarian TF cataloging has recently been undertaken.^37^ We searched the translated planarian transcriptome for genes encoding known DNA-binding domain (DBD) motifs using HMMER.^38,39^ This identified 1,317 transcripts representing 970 unique genes (Table S2). Many genes in this set were previously characterized. To classify uncharacterized genes, we performed BLAST against the human proteome. Genes with significant similarity to non-TFs were removed, resulting in 716 unique genes with domains resembling characterized DBDs or that have previously been identified in planarians (Table S3).

Within our DBD-containing catalog, 77 genes (11%) were previously classified as FSTFs and another 102 (14%) have been studied in other planarian contexts (Figure S1H). The remaining candidate TF genes consist of those with BLAST hits to human TFs (242 genes; 34%) and genes with no BLAST hit to any human protein (295 genes; 41%). Among known planarian FSTFs, homeodomains, zinc fingers, and forkhead domains collectively made up the majority of domains within gene products (63% total; 33%, 19%, and 11% respectively) (Figure 1H).

We calculated the expression enrichment of TFs for each major tissue type in neoblasts (Figure S1I). Previous work showed that all 118 *Caenorhabditis elegans* neuron classes can be delineated by unique homeodomain expression combinations.^40^ Most tissue-type progenitor classes had enriched expression for at least one homeodomain TF (Figure S1J). Overall, these findings indicate that neoblast classes can be delineated by their expression of TFs.

### Muscle cell-type diversity emerges in the S/G_2_/M neoblast stage

**Figure 2.**
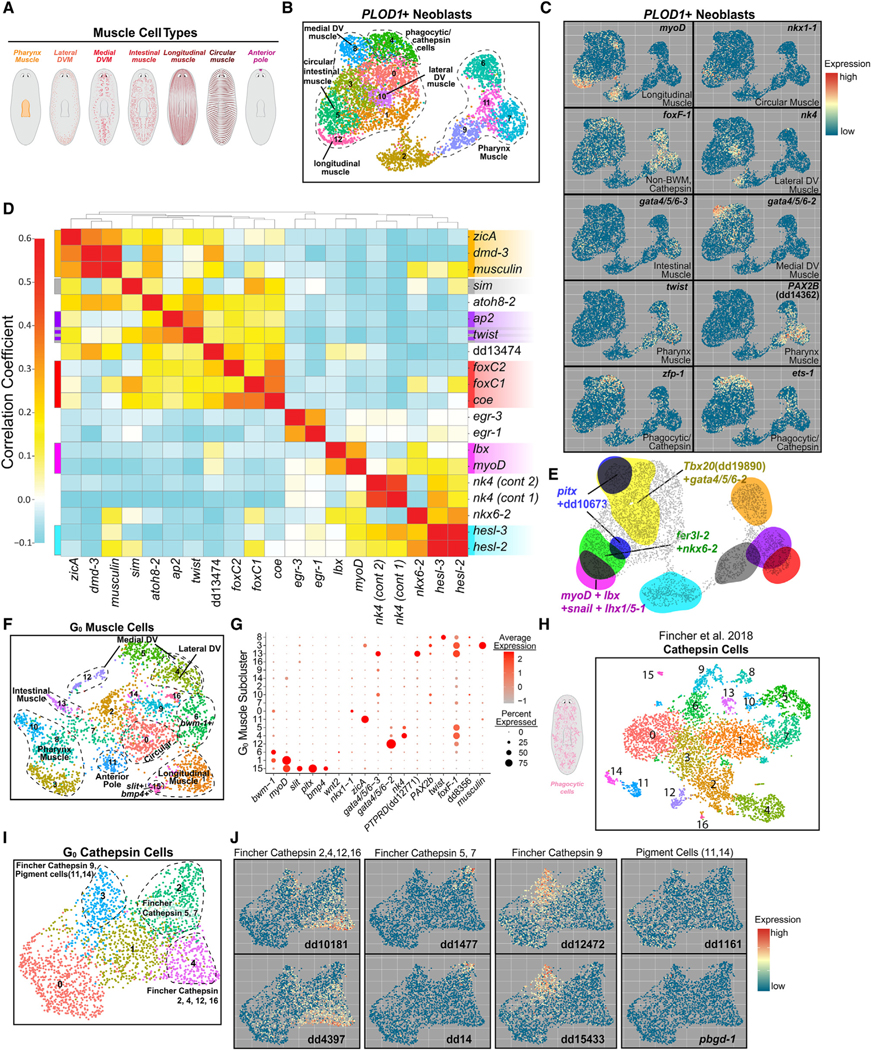
Cell-type transcriptome diversity is apparent in the S/G_2_/M neoblast stage for muscle but not for phagocytic/*cathepsin*+ cells (A) Planarian muscle cell types. (B) UMAP plot: subclustered *PLOD1+* S/G_2_/M neoblasts. (C) Expression of subtype-specific TFs in *PLOD1+* S/G_2_/M neoblasts. (D) Correlation analysis showing TF modules for muscle S/G_2_/M neoblasts. (E) TF-module expression domains for *PLOD1*+ S/G_2_/M neoblasts. Ensemble colors correspond to ensembles in (D) or are indicated separately. (F) UMAP plot showing subclustered G_0_ muscle cells. (G) Dot plot showing expression of muscle subtype markers across G_0_ muscle clusters. (H) Identified *cathepsin*+ populations from Fincher et al.^1^ (I) UMAP plot showing subclustered G_0_ *cathepsin*+ cells. (J) Expression of subtype-specific markers in G_0_ *cathepsin*+ clusters.

To compare the degree of specification in the S/G_2_/M and post-mitotic progenitor stages, we performed subclustering of cells by tissue type. We first examined the three clusters expressing *PLOD1*, which marks muscle and phagocyte/*cathepsin+* precursors.^1^ Planarians possess a diverse array of muscle subtypes with different roles in their physiology and in regulating regeneration (Figure 2A).^25,26,41–45^ Body-wall muscle (BWM) has anterior-posterior (AP)-oriented longitudinal fibers, circular fibers perpendicular to the AP axis, and diagonal fibers.^41^ Two dorsoventral muscle (DVM) fiber types (medial and lateral) connect dorsal and ventral regions.^26^ Supportive muscle surrounds the intestine and a separate pharynx muscle network exists. Finally, muscle-like anterior and posterior pole cells at animal ends organize pattern during regeneration.^5^ Each muscle subtype is specified by distinct TFs.^25,26^

Subclustering *PLOD1*+ S/G_2_/M cells identified 13 total subclusters (Figure 2B). Most had unique TF expression specifying different muscle subtypes, and almost all muscle cell types were represented by their own subcluster (Figure 2C). *gata4/5/6–2* (medial DVM^26^) and *nk4* (lateral DVM^26^) expression was enriched in cluster 8 and cluster 10, respectively (Figure 2C). *nkx1–1* (circular muscle^25^) and *gata4/5/6–3* (intestinal muscle^26^) were expressed in different regions of subcluster 5. *myoD* (longitudinal muscle^25^) was expressed in clusters 12 and 2. Subcluster 2 had unknown fate and had enriched expression of multiple TFs, including *hesl-3*, *hesl-2*, *musculin*, *myoD*, *nk4*, and *hnf4* (Figures 2C and S2A). Clusters 6, 7, 9, and 11 collectively expressed pharynx muscle markers (Figure 2C)^1^ and expressed separate TFs, including *musculin*, *dmd-3*, *ap2*, *coe*, *sim*, *foxC1*, and *zicA*. Anterior pole cells (expressing *foxD* and *prep*) were present, although not as a unique cluster (Figure S2A). *foxF-1* is expressed broadly across non-BW muscle^26^ and was expressed across multiple clusters, including neoblasts for pharynx muscle and other non-BW muscle^26^ (Figure 2C). Expression of positional control genes (PCGs), which constitute positional information and are primarily expressed in muscle cells,^45^ was detectable in different muscle-specialized neoblasts (Figure S2A). Finally, we used the TF catalog to define TF modules for muscle subtypes (Figures 2D and 2E). These data define transcriptomes and TF modules for S/G_2_/M cells with fates specified to essentially each of the different muscle subtypes of the animal.

We subclustered post-mitotic G_0_ muscle progenitors, revealing 17 clusters (Figure 2F). Most subclusters displayed enriched expression of cell-type-specific FSTFs and post-mitotic markers (Figures 2G and S2E). Cluster 4 and 12 represented DVM and had enriched expression of *nk4* and *gata4/5/6–2*. Cluster 13 represented intestinal muscle and was enriched in expression for *gata4/5/6–3* and the known intestinal muscle marker *PTPRD*.^26^ Clusters 0, 1, 6, and 15 expressed the BWM marker *bwm-1*, with cluster 0 enriched for circular fiber marker expression and clusters 1 and 15 enriched for longitudinal-fiber markers (Figure 2G). Cluster 15 also expressed the midline and dorsal PCGs *slit* and *bmp4*. Clusters 3, 8, and 10 represented pharynx muscle. Finally, a distinct cluster of anterior pole cells could be identified in cluster 11 based on the enriched expression of *foxD*, *prep*, and *zicA*, along with anterior PCGs (e.g., *sFRP-1*) (Figure S2E). These results are consistent with known muscle specification events that indicate distinct fates emerge from the earliest possible cellular stage (the neoblast) without intermediate branches of fate refinement.

### Lack of neoblast diversity of *cathepsin*+ cell populations

Subclustering of *PLOD1*+ S/G_2_/M cells also identified a single cell population (cluster 4) putatively specified for phagocytic/ *cathepsin+* cells (Figure 2B). Planarian phagocytic cells are composed of diverse cell types (at least 9) with phagocytic capacity and complex morphologies, including pigment and glial cells^1^ (Figure 2H; adapted from Fincher et al.^1^). *foxF-1* is broadly expressed across phagocytic cells and is required for specifying many of these cell types.^26^ *ets-1* promotes pigment cell specification,^14^ and its expression is broad throughout the phagocytic population as well.^1^ *hnf4* is also expressed in neoblasts expressing phagocytic cell markers.^1^ S/G_2_/M cells associated with the phagocytic fate had enriched expression of these three TF-encoding genes, along with *zfp-1* and *hunchback* (Figures 2C and S2A). Expression of these genes was broad throughout cluster 4, without overt substructure. Subclustering generated seven clusters that were not well separated and some of these cells expressed markers for phagocytic cell subtypes (Figures S2B–S2D). However, these markers largely had expression scattered broadly across subclusters.

Subclustering of post-mitotic phagocytic progenitors identified clusters enriched in cell-type-specific marker expression, consistent with these clusters representing progenitors for several of the known differentiated phagocytic cell subtypes (Figures 2I, J, and S2F). However, not all phagocytic cell-type diversity was apparent in the G_0_ post-neoblast progenitors either. These findings raise the possibility that diversification of *cathepsin*+ cell states might not occur until after the S/G_2_/M stage in post-mitotic progenitors, or was not clearly represented by distinct transcriptomes in the S/G_2_/M stage. These findings are in stark contrast with muscle progenitors, for which transcriptome heterogeneity matching the different classes of differentiated muscle was readily identified in the S/G_2_/M neoblast stage.

### Parenchymal progenitor fate diversity emerges in neoblasts

**Figure 3.**
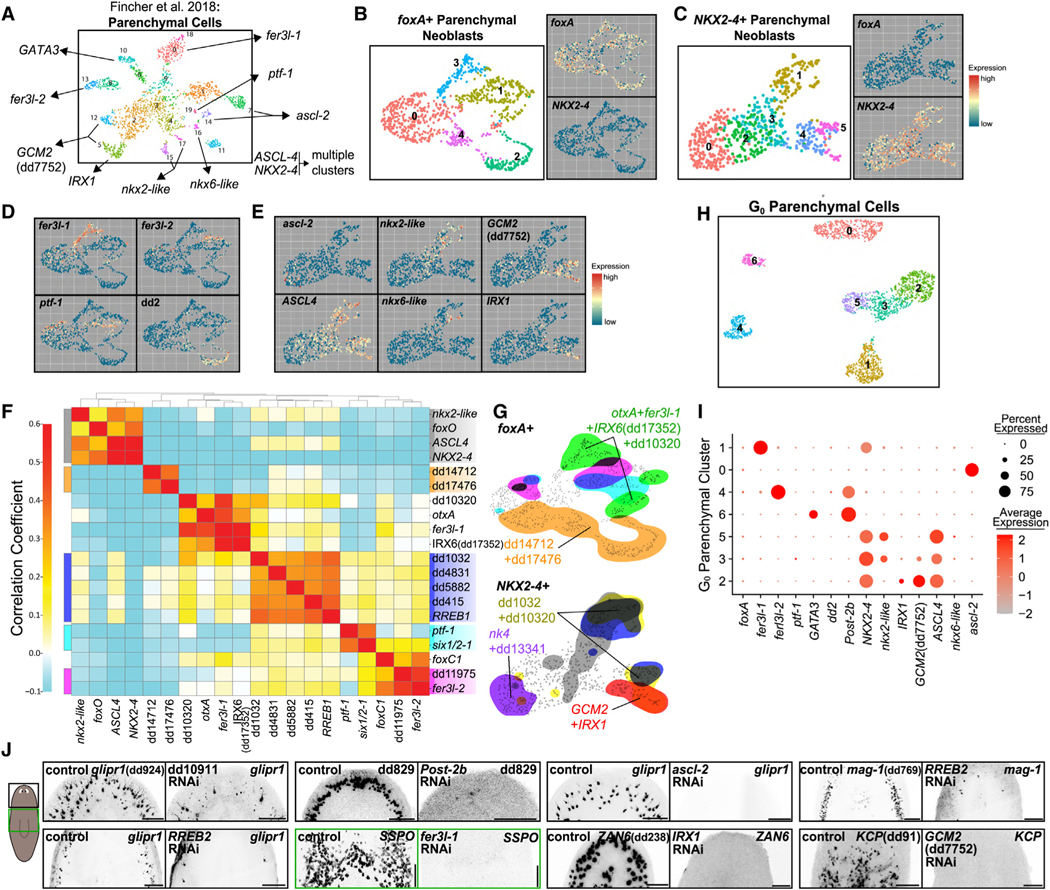
Parenchymal cell-type diversity emerges in neoblasts (A) Identified parenchymal populations from Fincher et al.,^1^ with enriched TF-encoding genes. (B and C) Subclustering of *foxA*+ and *NKX2–4+* S/G_2_/M parenchymal cells. (D and E) Marker gene expression across *foxA*+ and *NKX2–4* S/G_2_/M parenchymal cells. (F) Correlation analysis showing TF modules for parenchymal S/G_2_/M neoblasts. (G) TF-module expression domains for S/G_2_/M parenchymal cells. Ensemble colors correspond to TF ensembles in (F) or are indicated separately. (H) UMAP plot showing subclustered G_0_ parenchymal cells. (I) Dot plot: parenchymal subtype markers across G_0_ parenchymal clusters. (J) FISH: loss of parenchymal cell types following RNAi of subtype-specific TF-encoding genes. RNAi condition, top left; marker, top right. Head (black box) or trunk (green box) regions shown. Scale bars, 100 μm.

Parenchymal cells are a heterogeneous collection of cell types scattered within a mesenchymal tissue, and include gland cell types.^1,33,46^ Fate specification of these cells is poorly understood. Recent work identified 13 distinct differentiated parenchymal cell types with diverse spatial distributions^1^ (Figure 3A; adapted from Fincher et al.^1^). An additional eight putative transition states were identified of unclear neoblast or post-mitotic progenitor origin.

We found two TF-encoding genes, *RREB1* and *RREB2*, with enriched expression in mature parenchymal tissues and that were expressed in two S/G_2_/M neoblast clusters (Figure 1C). These clusters displayed mutually exclusive expression of the TFs *NKX2–4* and *foxA*. *foxA* is required for pharynx regeneration^10,23^ and a separate, unrelated *foxA+* cluster represented pharynx neoblasts (Figure 1C).

We separately subclustered *foxA*+ and *NKX2–4*+ 4C parenchymal neoblasts (Figures 3B and 3C). Multiple subclusters displayed enriched TF expression specific to distinct differentiated parenchymal subtypes (Figures 3D and 3E). *foxA*+ parenchymal neoblasts produced five subclusters. Cluster 3 expressed *fer3l-1*, marking putative progenitors for *SSPO* (dd_9342) differentiated cells.^1^ Cluster 1 expressed *fer3l-2*, marking putative progenitors for *mag-1+* cells.^1^ Cluster 1 also expressed *ptf-1*, but in a separate domain. *ptf-1* expression was enriched in putative progenitors for dd_8476 cells, a unique head parenchymal population.^1^ Clusters 2 and 4 expressed the non-TF-encoding dd_2 gene, which marks a parapharyngeal parenchymal population (parenchymal cluster 9 in Fincher et al.^1^). Correlation analysis identified several TF modules corresponding to identified parenchymal neoblast types (Figures 3F and 3G). Notably, this revealed two TF-encoding genes, dd_14712 and dd_17476, that were coexpressed in clusters 2 and 4 of the *foxA+* cluster. 4 of the 13 known parenchymal cell-types^1^ were represented within the *foxA+* S/G_2_/M parenchymal-specialized neoblast cluster.

Subclustering of *NKX2–4+* S/G_2_/M parenchymal neoblasts identified subclusters corresponding to the other differentiated parenchymal cells (Figure 3C). Cluster 5 expressed *IRX1* (Figure 3E). In prior data,^1^ *IRX1* expression was enriched in putative progenitors for differentiated *ZAN6+* (dd_238) cells (Figure 3A). Cluster 5 had enriched expression of *GCM2* (Figure 3E). In Fincher et al.,^1^ *GCM2* was expressed in putative parenchymal progenitors; a subset of these expressed *KCP* (dd_91) and another subset coexpressed *IRX1*. Finally, cluster 1 displayed enriched expression of *nkx2-like* and *nkx6-like* (*nkx2-like* expressed broadly and *nkx6-like* expressed in a cluster 1 subset). *nkx2-like* was expressed in several putative parenchymal progenitors, as well as three differentiated parenchymal clusters, including candidate progenitors for the dd_515 population (Figure 3A). Within the *NKX2–4+* S/G_2_/M parenchymal-cell-specialized neoblasts, a small fraction (primarily in cluster 0) expressed the TF-encoding *ascl-2* gene (Figure 3E). These results suggest that parenchymal cell diversity is generated in 10 populations of S/G_2_/M specialized neoblasts, grouped into two major categories: *foxA+* cells, subdivided into *fer3l-1+*, *fer3l-2+*, *ptf-1+,* and dd_2+ neoblasts; and *NKX2–4+* cells, subdivided into *IRX1+*, *GCM-2+*, *nkx2-like+*, *ascl-2+*, and *nkx6-like+* subsets.

To assess how subtype diversity was reflected in the G_0_ stage, we combined G_0_ post-neoblast parenchymal progenitors (1,996 cells) and performed subclustering, resulting in seven clusters (Figures 3H and S3A–S3D). One cluster (cluster 6) corresponded to the population of parenchymal cells marked by *GATA3* expression from Fincher et al.^1^parenchymal clusters 6 and 10 (Figures 3A and 3I). These cells were enriched in expression for *GATA3*, *pax6A*, *Post-2b*, *EVX1*, and *fer3l-3* (Figure S3A), similar to a progenitor state from Fincher et al.^1^ Low levels of *GATA3* and *Post-2b* were present sparsely in one subcluster of parenchymal neoblasts, which may be their precursor (Figure S3E).

We tested the functional requirement for parenchymal FSTFs in specifying parenchymal subtypes using RNA interference (RNAi) (Table S4). Because of cell turnover, RNAi of a TF-encoding gene required for fate specification can lead to steady depletion of the specified cell type.^5^ Furthermore, amputation requires *de novo* cell-type formation in blastemas. RNAi animals were subjected to head and tail amputation and were fixed after regeneration (>10 days post amputation [dpa]). Inhibition of many TFs led to reduced parenchymal cells expressing cell-type-specific markers. This could reflect depletion of those cells or loss of marker expression. In many cases, RNAi resulted in fewer cells robustly expressing the marker gene used, rather than lower levels of marker gene expression throughout the cell population, consistent with cell loss (Figures 3J and S4A–S4G). Furthermore, cell absence was typically observed in both blastemas and uninjured tissues in these animals, indicating requirements were not specific to regeneration (Figures 3J and S4A–S4G). *IRX1* RNAi and *GCM2* RNAi led to depletion of *ZAN6+* (dd_238) and *KCP+* (dd_91) parenchymal cells, respectively. RNAi of *fer3l-1* led to the depletion of *SSPO* (dd_628+)-expressing cells (Figures 3J and S4E). *ascl-2* RNAi eliminated cells expressing *glipr-1* (dd_924), as did RNAi of dd_10911, which encodes a zinc-finger protein (Figures 3J, S4A, and S4C). We also found that, although *RREB1* and *RREB2* expression globally marked 4C parenchymal neoblasts, only *RREB2* was functionally required for forming any of the parenchymal subtypes tested (Figures 3J and S4D). Finally, RNAi of *Post-2b*, which was expressed in G_0_ cell-specific cluster 6, resulted in loss of dd_829-expressing parenchymal cells (Figures 3J and S4B). Overall, these results identify numerous FSTFs associated with new specialized neoblast classes and show that specification of most-to-all of the cell-type diversity of parenchymal cells is generated in the 4C stem cell state.

### Specification of fate in progenitors of the nervous system

**Figure 4.**
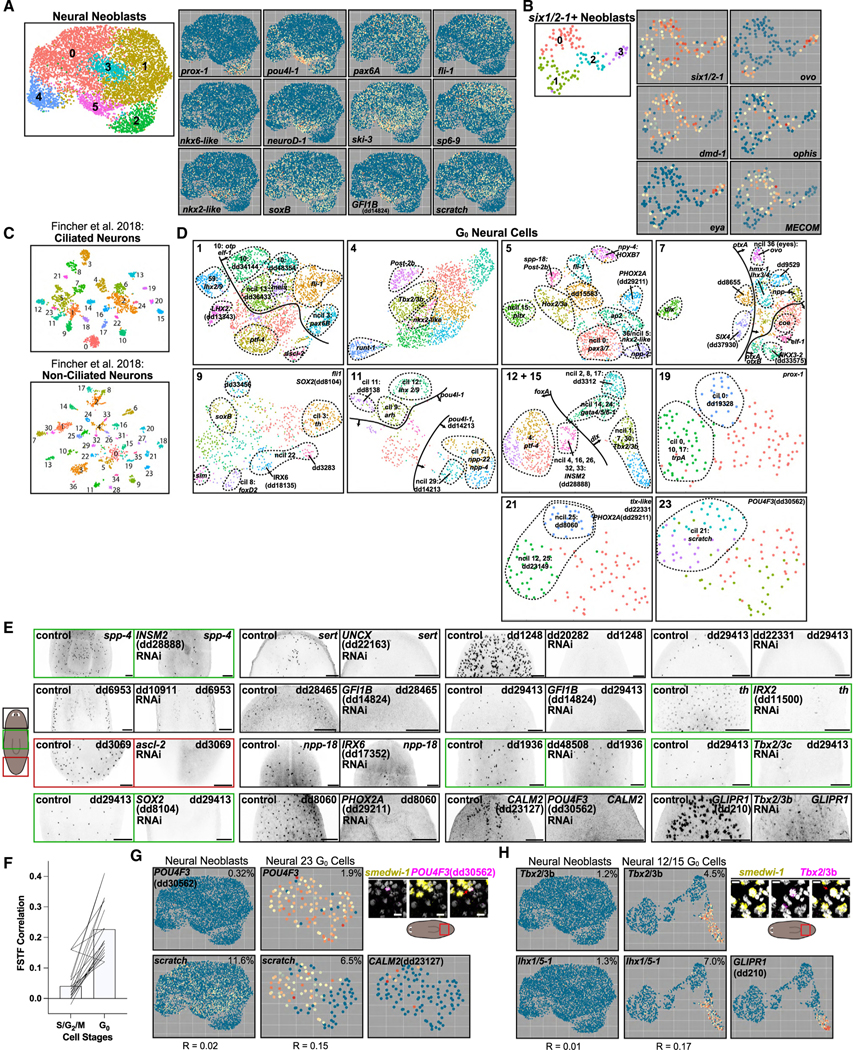
More cell states are present in neural post-mitotic progenitors than in S/G_2_/M neoblasts (A) UMAP plot: subclustered neural S/G_2_/M cells and neural transcription factor (TF) expression. (B) UMAP plot: subclustered *six1/2–1*+ S/G_2_/M cells and expression of genes encoding TFs and germline markers. (C) Identified neuron clusters from Fincher et al.^1^ (D) UMAP plot: subclustered G_0_ neurons. Numbers in upper left refer to G_0_ main cluster number. Enriched TFs labeled for several subcluster regions. ncil (non-ciliated) and cil (ciliated) refer to non-ciliated and ciliated subclusters from Fincher et al.^1^ Numbers not preceded with a label refer to subclusters from “all neural” of Fincher et al.^1^ (E) FISH images showing loss of neural cell types following RNAi of subtype-specific TF-encoding genes. RNAi condition, top left; marker, top right. Head (black box), trunk (green box), or tail (red box) regions shown. Scale bars, 100 μm. (F) Expression correlation values among neural TF pairs between S/G_2_/M and G_0_ neural cells. Lines connect the same TF pairs between cell states. (G) Expression of *POU4F3* and *scratch* in S/G_2_/M neural cells and G_0_ cells. *CALM2* expression in G_0_ neural cells. FISH: *smedwi-1* and *POU4F3* coexpression. (H) Expression of *Tbx2/3b* and *lhx1/5–1* in neural S/G_2_/M and G_0_ cells. *GLIPR1* expression in G_0_ neural cells. FISH: *smedwi-1* and *Tbx2/3b* coexpression.

The planarian nervous system contains more cell-type diversity than any other tissue.^1^ S/G_2_/M neoblast clustering revealed two populations containing neural progenitors: one labeled “neural” and the other unified by *six1/2–1* expression (Figure 1B). We subclustered these populations independently (Figures 4A and 4B). Two *six1/2–1+* subclusters were eye progenitors, expressing eye FSTFs such as *ovo* and *eya* (Figure 4B). *six1/2–1+* subclusters also contained cells expressing the TF-encoding *MECOM* and *dmd-1* genes; *dmd-1* is expressed in somatic support cells for the germline.^47^ Subclustering of the 4C neural population (6,526 cells) revealed six main clusters, expressing known neural FSTFs (Figure 4A). Cluster 2 had enriched expression of neural FSTFs, including *nkx6-like*^23^ and *prox-1*. Cluster 5 had enriched expression of the neural FSTFs^11,12,48^ *pou4l-1*, *neuroD-1*, and *nkx2-like*. Most other known neural FSTFs^18,23^ were expressed broadly across multiple subclusters, including *pax6A*, *ski-3*, *fli1*, *sp6–9*, and *scratch*. The neural 4C cluster was the primary *tgs-1*^*+*^ cluster, which was hypothesized to mark a specific pluripotent cluster in Zeng et al., but was also shown to mark neural-specified neoblasts in Raz et al. (Figure S5A).^9,32^ Seventy-eight percent of the cells in this *tgs-1*^*+*^ cluster expressed one or more of 18 neural FSTFs (Figures S5B–S5D).

Correlation analysis among neural neoblasts identified several TF modules (Figures S6A and S6B). A TF-encoding gene with similarity to human *GFI1B* (dd_14824) had correlated expression with known neural FSTFs *pou4l-1* and *neuroD-1* (Figure S6A). *GFI1B* was expressed in ciliated neuron populations from Fincher et al.,^1^ such as clusters 11 and 18 (marked by dd_28465 and dd_29413, respectively; Figure 4C). *GFI1B* RNAi led to loss of both of these differentiated neuron populations (Figure 4E). *ascl-2* (dd_14753) and dd_14712, which encodes an ETS-domain TF, had correlated expression with each other and the neural FSTF *prox-1* (Figure S6A). These genes were expressed in a subset of neurons expressing dd_3069, and RNAi of *ascl-2* resulted in the loss of these cells (Figure 4E). Using Fincher et al.^1^ scRNA-seq data, we found that *IRX6* was expressed in a unique subset of non-ciliated *npp-18*+ neurons. *IRX6* RNAi resulted in loss of *npp-18*+ cells (Figure 4E). *INSM2* (dd_28888) had modestly correlated expression with neural FSTFs, including *tcf/lef* and *otxB* (R = 0.08 and R = 0.12). *INSM2* was expressed in a subset of neurons expressing *spp-4*, and *INSM2* RNAi resulted in reduction of *spp-4*+ cells (Figure 4E). These results identify novel neoblast classes for mature neural subtypes and FSTFs that delineate them.

To study neural fate in neoblast progeny, we subclustered post-mitotic neural progenitors (Figure 4D). A total of 77 G_0_ neural progenitor clusters were identified, many more than the seven neural X1 clusters identified. In Fincher et al.,^1^ at least 70 differentiated neuron clusters were identified (Figure 4C). There is therefore a similar scale of identified heterogeneity in G_0_ neural progenitors and mature neurons. We were able to assign 37 subclusters as candidate G_0_ neural progenitors for distinct differentiated neural subtypes based on the shared unique marker expression (Figure 4D). An additional 30 subclusters could not readily be connected to a differentiated cluster from Fincher et al.^1^ but displayed expression of unique markers. Therefore, in total, at least 67 post-mitotic neural progenitor states were identified, with their transcriptomes and associated FSTFs defined.

Because the number of neural G_0_ progenitor classes was far greater than the number of neural S/G_2_/M neoblast clusters, it is possible that neural fate heterogeneity increases in post-mitotic stages. To explore this possibility further, we first assessed the expression of neural FSTFs expressed in G_0_ subclusters within S/G_2_/M neoblasts. Many of these genes were expressed in a similar number of both neural neoblasts and their post-mitotic descendants. However, although they were expressed in specific subclusters within G_0_ cells, they were not expressed in a specific subcluster (or region of UMAP space) in S/G_2_/M neoblasts (Figure S6C). This differed from neoblasts for other cell types, such as muscle, where cells expressing FSTFs clustered together. This could indicate that neural neoblasts are more homogeneous and less defined than G_0_ neural progenitors, even when they express neural FSTFs. We next asked if FSTFs that are coexpressed in G_0_ neural progenitors tended to also be coexpressed in neoblasts. It was previously shown that *pitx* and *lhx1/5–1* specify serotonergic neuron progenitors,^13,17^ and these TFs were coexpressed in G_0_ cells (Figure S6D). However, despite being individually expressed in S/G_2_/M neoblasts, they were not coexpressed and their gene expression correlation was lower than in G_0_ progenitors (R = 0 vs. R = 0.29). We also found a TF-encoding gene, *UNCX*, that had highly correlated expression to *pitx* in post-mitotic progenitors. *UNCX* was also expressed in a relatively similar number of S/G_2_/M neoblasts but expression was less correlated to that of *pitx* compared to in G_0_ progenitors (R = 0.07 vs. R = 0.41; Figure S6E). Another example G_0_ progenitor population (cluster 7; Figure 4D) expressed *otxA* and *otxB*, yet these genes were uncorrelated in S/G_2_/M neoblasts (R = 0.01 in neoblasts vs. R = 0.33 in G_0_ progenitors; Figure S6F). Among pairs of correlated TFs identified in G_0_ neural progenitors, many showed a similar increase in correlation from neoblast to post-mitotic progenitor (Figures 4F; Table S5). This indicates that some neural neoblasts did not express the full complement of TFs that define them in the post-mitotic state.

We next identified novel FSTFs required for the presence of neural populations (Figures S7A–S7H and S8A–S8G). *PHOX2A*, which encodes a paired-like homeodomain TF, defined a unique G_0_ neural progenitor population with an expression signature corresponding to dd_8060 peripheral neurons (Figure S8E). *PHOX2A* RNAi eliminated dd_8060+ neurons (Figure 4E). *POU4F3* was specifically expressed in a G_0_ neural progenitor population corresponding to *CALM2+* (dd_23127) neurons (ciliated cluster 21 from Fincher et al.^1^) and *POU4F3* RNAi led to loss of *CALM2*+ neurons (Figures 4E, 4G, and S8F). *PHOX2A* was expressed in 4C neural neoblasts, but in a scattered pattern. Similarly, *POU4F3* was also expressed in 4C neural neoblasts by sequencing data and by fluorescence *in situ* hybridization (FISH) (Figure 4G). *POU4F3* was coexpressed with the known neural FSTF *scratch* in G_0_ cells, but not in neoblasts. The neural population represented by *CALM2* expression was shown to be regulated by the FSTF *soxB1–2*.^21^ In the Fincher et al.^1^ atlas, *soxB1–2* is expressed in the cluster marked by *CALM2* but also in several other clusters. *Tbx2/3b* was expressed robustly in a G_0_ neural progenitor population associated with *GLIPR1*+ (dd_210) peripheral neurons (Figure 4H). *Tbx2/3b* RNAi resulted in loss of *GLIPR1+* cells, establishing *Tbx2/3b* as an FSTF required for this neural population (Figure 4E). *Tbx2/3b*+ neoblasts were identified in uninjured animals by FISH (Figure 4H), but coexpression of *Tbx2/3b* and *lhx1/5–1* (another TF-encoding gene enriched in this population) was relatively rare in 4C neoblasts compared to post-mitotic progenitors. An additional eight examples of FSTF RNAi – cell-type ablation were found. These included (1) *IRX2* RNAi and loss of tyrosine hydroxylase (*th*)-positive dopaminergic neurons, (2) *UNCX* RNAi and loss of *sert*-positive serotonergic neurons, (3) dd_10911 RNAi (encoding a zinc-finger protein) and loss of dd_6953-positive peripheral neurons, and (4) RNAi of three TFs that each led to loss of dd_29413-positive ciliated neurons (including *Tbx2/3*c and a *SOX2* gene) (Figures 4E, S7A–S7H, and S8A–S8G).

Together, these analyses demonstrate that numerous unique neural states were identified in the G_0_ post-mitotic progenitor data and that they could be associated with mature neuron types through TF and marker expression similarity. For several of these, FSTFs were required for presence of the mature cell type. The progenitors for many of these cell types were not overtly present in neural-specialized neoblasts as unique subclusters or through expression of their characteristic TF modules, consistent with fate diversification or maturation occurring post-mitotically. The neural population of 4C neoblasts will be an intriguing target for continued investigation to unravel the underpinnings of neural fate choice in neoblasts.

### Expansion of cell-type diversity post-mitotically

As described above, neoblasts for neural and *cathepsin+* populations contained far fewer identifiable cell classes than their differentiated counterparts. To further explore the possibility of fate diversification occurring in post-mitotic stages for these tissues, we compared neoblast and post-neoblast progenitor stages for both classes. Both neural and phagocytic/*cathepsin+* tissues had more post-mitotic clusters compared to neoblasts. The subclustering results with neural neoblasts and neural post-mitotic progenitors were robust to clustering (Louvain and Leiden) and visualization methods (UMAP and pairwise controlled manifold approximation [PaCMAP]), and the number of principal components used (Figures S9A–S9C). We used k-means clustering of cells in these classes using the top principal components in the dataset as another way to assess cell-type number in the data. To measure the number of clusters/ states in the data we used the Ratkowsky index, which is a measure of the quality of clustering. By using the Ratkowsky index for a variable number of cell-state identities, the number of groups in each cell state (4C and post-mitotic) that best explains the differences between cells can be found. For neural progenitors, the maximal Ratkowsky index was associated with more groups in post-mitotic progenitors compared to neoblasts, consistent with a greater number of cell states emerging post-mitotically (Figure S9D). *cathepsin+*-fated progenitors also showed an increase in group number in post-mitotic progenitors compared to 4C neoblasts, with a more modest difference compared to neural states. By contrast, muscle-fated cells had similar numbers of groups in the neoblast and post-mitotic progenitor datasets. This analysis lends further support to the idea that some cell types continue to diversify after initial specification as neoblasts.

We also used URD, a transcriptome pseudotime trajectory analysis method, to seek corresponding specialized neoblasts for neural and muscle-specialized post-mitotic progenitors. URD does not generate cell lineages, but was used to assess possible transcriptome trajectories between stages. For muscle-fated cells, URD produced pseudotime trajectories with terminal segments corresponding to most of the muscle types identified previously (Figures S10 and S11). Terminal segments had a mixture of specialized neoblasts and post-mitotic progenitors that we previously associated using markers. For neural-fated cells, URD generated terminal segments corresponding to different broad neural post-mitotic progenitors (Figures S12 and S13). However, these terminal segments had far fewer specialized neoblasts than did muscle segments (Figure S12I). Neural-specialized neoblasts and post-mitotic progenitors also had less overlap in their pseudotime distributions than did muscle neoblasts and post-mitotic progenitors, possibly indicating less transcriptional similarity and further consistent with greater transcriptome diversification post-mitotically in the case of neural fates (Figures S12H).

### A *six1/2–1+* cluster associated with germline niche cells

**Figure 5.**
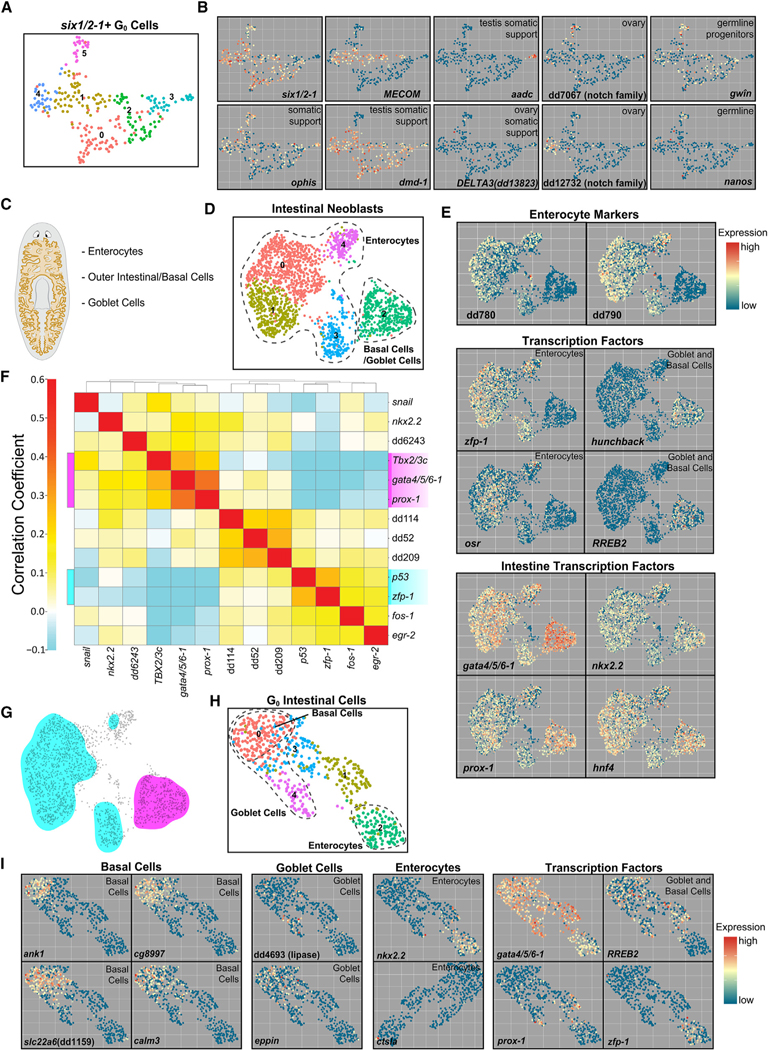
Cell transcriptomes identify two distinct S/G_2_/M intestinal neoblast states and germline support cell progenitors (A) UMAP plot: subclustered *six1/2–1+* germline-associated G_0_ cells. (B) Expression of germline-associated markers in *six1/2–1+* G_0_ cells. (C) Planarian intestinal cell types. (D) UMAP plot: S/G_2_/M intestinal cell subclustering. (E) Expression of intestinal markers in intestinal S/G_2_/M cells. (F) Correlation analysis showing TF modules for intestinal neoblasts. (G) TF-module expression domains for intestinal S/G_2_/M cells. Ensemble colors correspond to ensembles in (F). (H) UMAP plot: subclustering of G_0_ intestinal cells. (I) Expression of intestinal subtype markers in intestinal G_0_ cells.

Post-mitotic progenitor cluster 17 (Figure 1F) had enriched expression of *six1/2–1*, *dmd-1*, and *MECOM* and likely represents descendants of *six1/2–1*^*+*^ S/G_2_/M neoblasts that expressed these genes (Figures 5A and 5B). These cells did not express neural markers (Figure S14A). *dmd-1* is expressed in testis gonad support cells in sexually reproducing *Schmidtea mediterranea*.^47^ These cluster 17 cells also expressed *ophis*, *aadc*, and *laminA* (Figure 5B), other genes that are expressed in the sexual accessory cell types in sexual *S. mediterranea*.^49–51^ Some of these genes are also known to be expressed in asexual *S. mediterranea*, which possess germ cells but not mature gametes.^52^ Two *notch*-family genes were found to be expressed in the ovaries in sexual planarians^50^ and were also expressed in subclusters of these G_0_ cells. The gene referred to as *notch2* (dd_7067) in Khan et al. 2022 was expressed in a subcluster devoid of *dmd-1* expression, as expected given this gene is expressed in ovaries and *dmd-1* is expressed in the testis. These results suggest this cluster of cells likely contains progenitors for the somatic support cells for the germline in asexual planarians.

### Distinct S/G_2_/M intestine states correspond to mature intestinal subtypes

The planarian intestine is composed of three main cell types: enterocytes, basal/outer intestinal cells, and secretory goblet cells dispersed within the enterocyte layer^1,53^ (Figure 5C). Several FSTFs regulate specification of intestine neoblasts, including *gata4/5/6–1*, *nkx2.2*, *prox-1*, and *hnf4*.^3,27,54,55^ Intestinal neoblasts have largely been considered a homogeneous specialized neoblast population, despite mature intestine cell-type heterogeneity. Clustering of S/G_2_/M neoblasts identified two distinct intestinal neoblast populations (Figure 1B). We combined both clusters and performed subclustering, generating five subclusters (Figure 5D). Four clusters (subclusters 0, 1, 3, and 4) were identified as putative enterocyte precursors based on the expression of enterocyte-specific markers (Figure 5E). These clusters were also enriched in the expression of the TFs *zfp-1* and *osr*, FSTFs not previously known to be associated with intestine neoblasts. *zfp-1* has a well described and required role in epidermal neoblast specification and is expressed broadly in epidermal-specialized neoblasts.^27^ Cells of subcluster 2 did not express *zfp-1* and *osr* but were characterized by expression of *hunchback* and *RREB2*. *RREB2* was previously shown to be expressed in goblet and basal intestine cells.^53^ Correlation analysis identified TF modules that associated broadly into two main groups: the enterocyte clusters and subcluster 2 (Figures 5F and 5G). *zfp-1* and *p53*, which are associated with the epidermal lineage, were coexpressed in enterocyte neoblasts, but another epidermal TF (*soxP-3*) was not strongly expressed (Figure S14B). Subcluster 2 neoblasts coexpressed the known FSTFs *gata4/5/ 6–1* and *prox-1*, as well as *Tbx2/3*c (Figures 5F and 5G).

In post-mitotic G_0_ cells, two clusters of apparent intestinal progenitor cells existed (Figures 1F and 1G), corresponding to either enterocytes or basal/outer intestinal cells. We pooled these cells and subjected them to subclustering, revealing five subclusters (Figure 5H). The presumptive G_0_ enterocyte progenitors expressed *zfp-1* and *osr* and enterocyte-specific markers, similar to enterocyte neoblasts (Figures 5I and S14C). The presumptive G_0_ basal/outer intestinal cell progenitors had transcriptional similarity to subcluster 2 of the 4C-intestine neoblasts (*zfp-1*-, *osr*-, *RREB2*+), suggesting that subcluster 2 of the 4C intestine neoblasts represents outer-intestinal-cell-specialized neoblasts (Figures 5I and S14C). These clusters also expressed some goblet cell markers, indicating that they might give rise to basal and goblet cells (Figure 5I). Correlation analysis among intestine neoblasts showed that *zfp-1* is negatively correlated with both *RREB2* and *hunchback*, supporting the hypothesis that *zfp-1+/osr+* neoblasts and *RREB2+/hunchback+* neoblasts define distinct neoblast states (Figure S14D). Despite the fact that all intestinal neoblasts share expression of many canonical intestine FSTFs (e.g., *gata4/5/6–1*, *nkx2.2*, *prox-1*, and *hnf4*), these data indicate intestinal neoblasts are heterogeneous and generate cell-type diversity through at least two broad subtypes.

### Neoblast fates for the epidermis

**Figure 6.**
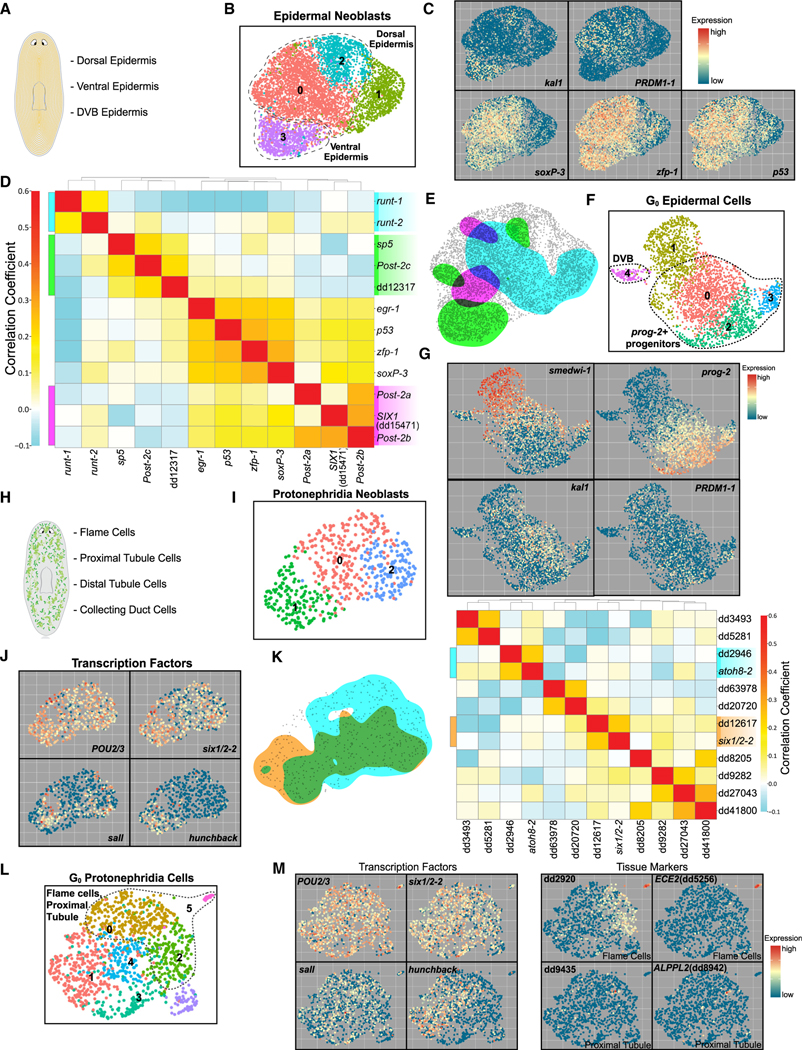
Diversification of epidermal cells arises from distinct neoblast subtypes, whereas protonephridia diversity arises from a common neoblast pool (A) Planarian epidermis cell types. (B) UMAP plot: S/G_2_/M epidermal cell subclustering. (C) Expression of epidermal subtype markers in epidermal S/G_2_/M cells. (D) Correlation analysis showing TF modules for epidermal neoblasts. (E) TF-module expression domains for epidermal S/G_2_/M cells. Ensemble colors correspond to ensembles in (D). (F) UMAP plot showing subclustering of G_0_ epidermal cells. (G) Expression of epidermal markers in epidermal G_0_ cells. (H) Planarian protonephridia cell types. (I) UMAP plot: subclustering of S/G_2_/M protonephridia cells. (J) Expression of protonephridia FSTFs in protonephridia S/G_2_/M cells. (K) Left: TF-module expression domains for protonephridia S/G_2_/M cells. Right: correlation analysis showing TF modules for S/G_2_/M protonephridia cells. (L) UMAP plot: G_0_ protonephridia cell subclustering. (M) Expression of protonephridia subtype-specific markers in protonephridia G_0_ cells.

The epidermis originates from neoblasts called zeta neoblasts, which produce post-mitotic progeny that transit through maturation stages with distinct gene expression stages.^27,56–58^ Dorsal and ventral epidermis are functionally distinct, and a unique population also exists at the dorsal-ventral (DV) animal boundary^57,59^ (Figure 6A). Importantly, dorsal and ventral identities originate in zeta neoblasts, marked by the expression of *PRDM1–1* and *kal1*, respectively.^57^ Subclustering of S/G_2_/M epidermal neoblasts identified unique clusters corresponding to dorsal and ventral precursors (Figure 6B). A third cluster displayed lower expression of canonical epidermal FSTFs (*soxP-3*, *p53*, and *zfp-1*) (Figure 6C). TF-correlation analysis identified modules that correspond to ventral and dorsal epidermal clusters (Figures 6D and 6E). An additional module had enriched expression of *Post-2a* and *Post-2b*, TFs previously found to be expressed in the mature DV boundary epidermis.^57^ This indicates that the epidermal DV boundary fate likely arises in neoblasts, not later in G_0_ progenitors where expression of the previously identified DV boundary FSTF *tlx-1* first arises.^57^ Subclustering of G_0_ epidermal progenitor cells identified dorsal and ventral epidermal progenitors, although these G_0_ cells clustered primarily by canonical epidermal lineage progression markers, such as *prog-2*, rather than dorsal/ventral markers (Figures 6F and 6G). These progenitors represented many cells expressing the early progression marker *prog-2* but few cells that expressed *agat-3*, a marker for a later stage of maturation (Figure S14E). An offshoot G_0_ progenitor subcluster (cluster 4) consisted of cells expressing TFs in the DV boundary TF module.

### Neoblast fates for the protonephridia

The planarian protonephridia functions as a waste excretion and osmoregulatory system that includes flame cells, proximal and distal tubule cells, and collecting duct cells^22,60,61^ (Figure 6H). Several TFs are important for protonephridia formation.^22^ Subclustering of S/G_2_/M protonephridia neoblasts revealed a seemingly homogeneous cell population (Figure 6I). Protonephridia FSTFs such as *POU2/3*, *six1/2–2*, and *sall* were expressed throughout these cells (Figure 6J). *hunchback*, an FSTF required for forming all protonephridia subtypes, displayed enriched expression primarily in cluster 1. Correlation analysis between protonephridia neoblasts identified modules of correlated TFs that did not mark distinct regions of cells within UMAP space, potentially indicating these modules are expressed broadly in protonephridia neoblasts (Figure 6K).

Subclustering of post-mitotic G_0_ protonephridia progenitors revealed a small number of clusters and regions corresponding to nephridia cell types (Figure 6L). Cluster 5, along with a small population of cells in regions of clusters 0 and 2, was positive for the flame cell markers dd_2920 and *ECE2* (dd_5256) (Figure 6M). A small region of cluster 0 expressed proximal tubule cell markers such as dd_9435 and *ALPPL2* (dd_8942). These results support a previously proposed model in which protonephridia subtypes emerge from a common pool of 4C neoblasts defined by expression of several FSTFs^22^ and suggest that cell-type diversity for these cells emerges later in post-mitotic states.

## DISCUSSION

Generating cell-type diversity is a foundational task not only for animal development but also in adult homeostasis and regeneration. Regeneration poses a number of unique challenges compared to development, and generating cell-type diversity might therefore involve different mechanisms in these contexts. First, cell-fate specification must be tailored to the identity of unpredictable missing tissues rather than initiating from a fixed starting point. Second, fate specification can occur in adult progenitors existing within organized, mature tissues, presenting different environments for influencing fate. Finally, although amplification of cells prior to differentiation is critical in development, wound sites in principle could have many progenitors from early time points. Planarians generate every adult cell type (~125 types) in adulthood during tissue turnover and in regeneration from neoblast stem cells.^1,5,33^ Planarians are thus attractive for uncovering solutions to the challenge of generating large-scale cell-type diversity in an adult context. Systematic identification of fate choices and the TF expression signatures associated with fate choices in neoblasts and post-mitotic progenitors will help understand the logic of fate choice in a regenerative context.

We utilized scRNA-seq on 4C neoblasts and 2C post-mitotic progenitors during regeneration to generate an atlas of stem cell fates and TFs that define them. Neoblasts have been proposed to exist in a hierarchy with a particular pluripotent class;^32^ other work has suggested a less hierarchical model in which specialized neoblasts can produce neoblast progeny that can choose a new fate in subsequent divisions, retaining potency.^9^ This work did not address lineage structure. 4C neoblasts were clustered into classes that corresponded to the major tissue types of the animal, and there was no clear unspecialized cluster. Other recent work shows that neoblasts choose fate in highly intermingled neighborhoods, lacking clear structure.^62^ Forty-six neoblast clusters/subclusters were identified in total, indicating these stem cells possess mechanisms to select between a large number (~40 or more) of possible fate options. The TF atlas of cell fates described in this work presents a wealth of new TFs and neoblast states that can be investigated to unravel the mechanisms by which neoblasts choose among myriad possible paths. Some of these TFs might control specific aspects of progenitor or differentiated cell physiology, which can also be studied using this atlas. In total, 129 separate clusters of post-mitotic progenitors were identified, similar to the estimated total number of planarian cell types. We were able to connect the majority of these progenitors to known differentiated states and to annotate the TF expression signatures of each of these progenitor types (TF atlas: Table S6). For many of these cell states and FSTFs that had not been previously investigated, we utilized RNAi to establish a requirement of the FSTF for regeneration and maintenance of the predicted target differentiated cell type.

**Figure 7.**
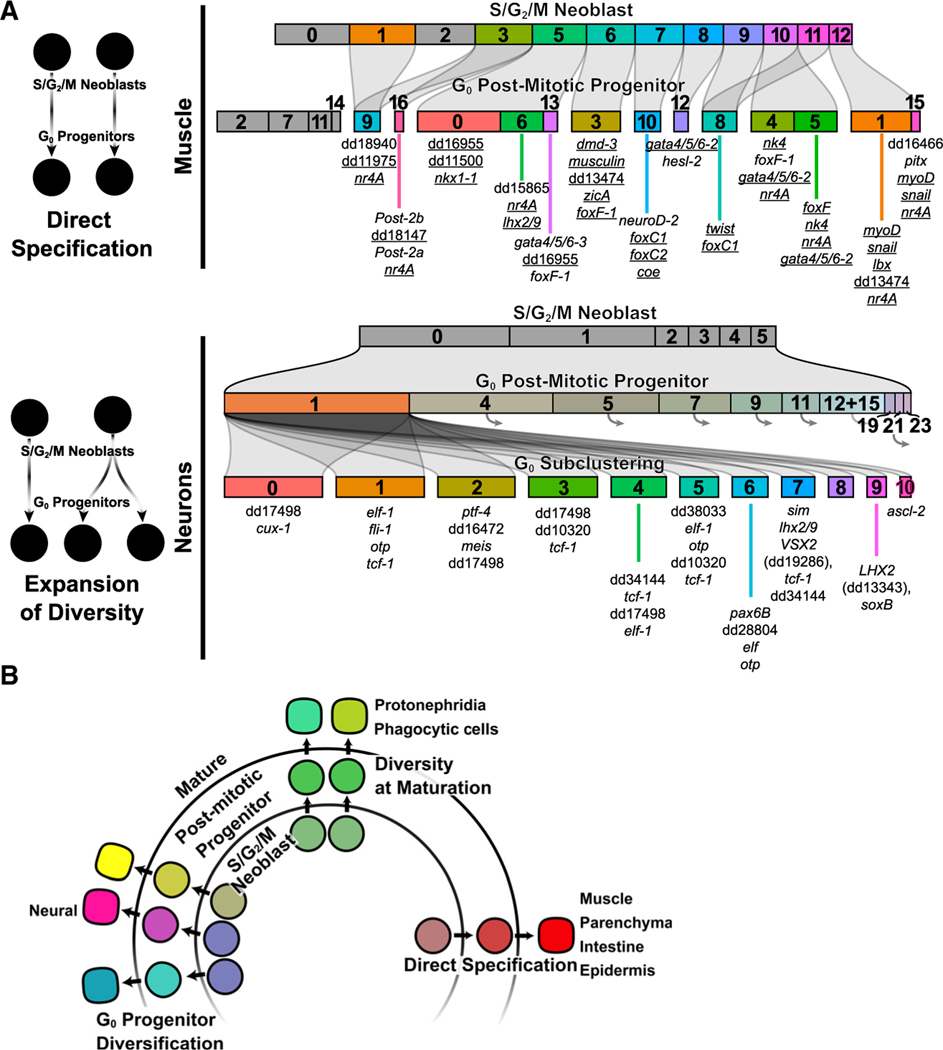
A transcription factor atlas of stem cell fate identifies organization principles used to generate cellular diversity in planarians (A) An atlas of neoblast and G_0_ post-mitotic progenitor cell types and their transcription factor (TF) signatures was used to connect G_0_ cell-type identity to neoblast identity. Cluster numbers correspond to UMAP subcluster identities. Connections between neoblast and G_0_ subclusters correspond to matching fate identities. TF signatures for fate paths are denoted below the G_0_ clusters. Underline denotes TF is expressed in corresponding neoblast and G_0_ cells. (B) Cell-type diversity emerges at different stages across planarian tissues. For some tissues (muscle, parenchyma, intestine, epidermis), fate diversification occurs at the earliest possible step, in the neoblasts. For other tissues, such as protonephridia and phagocytic cells, transcriptomic fate diversification has not emerged by the neoblast stage or fully by the G_0_ stage and likely occurs later. For the nervous system, substantial transcriptome diversification occurs by the post-mitotic G_0_ stage.

This work combined with prior studies on neoblast specialization identifies distinct principles for generating cell-type diversity across tissues (Figures 7A and 7B): within muscle and parenchymal cells, distinct transcriptional programs involving unique FSTF expression signatures demonstrate diversity is established in the 4C neoblast state, and these states are maintained into post-mitotic progenitors and differentiated cells. For intestine cell-type formation, all intestinal 4C cells are united by the expression of some major FSTFs, and exclusive expression of other FSTFs distinguish specialized neoblasts for distinct intestinal cell types (although it was difficult to conclusively separate basal and goblet fates because many currently known markers are shared between them, and many distinct markers for these cells were not expressed in corresponding specialized neoblasts). The epidermis is similar to these other tissues, with dorsal and ventral epidermal states apparent in 4C cells, as previously suggested using the markers *prdm-1* and *kal-1*,^57^ along with newly identified neoblasts for the DV boundary. These four tissues all represent cases of fate diversification at the earliest possible step: in the neoblasts.

4C neoblasts specialized for protonephridia and phagocytic/*cathepsin+* cells were apparent but not overtly heterogeneous, lacking clear cell-type-specific marker signatures, and were not composed of cell groups with multiple unique FSTF signatures. This raises the possibility that generation of cell-type diversity for these tissues could arise from a common pool of 4C precursors that diversifies in the G_0_ stage, as was previously proposed for the protonephridia^22^ (Figure 7B). Other molecular and functional studies will be important for continued assessment of this possibility.

Finally, neural differentiation involves heterogeneity in the 4C neoblast state, but this heterogeneity was limited when compared to the large degree of heterogeneity of mature neuron types. Several known neural FSTFs displayed enriched expression in a few common subclusters, whereas others were broadly expressed throughout the population, and others were rare and scattered in their expression. By contrast, substantial transcriptome heterogeneity was readily apparent for neural progenitors in the post-mitotic (post-neoblast) state in the form of many distinct cell-type-specific clusters. The fate choice for many mature neural cell types could be mapped onto these post-mitotic progenitors. These progenitors were distinguished by their expression of unique TFs (Figure 7A), many of which were not robustly coexpressed in neural neoblasts. Neural neoblasts and their post-mitotic descendants are therefore attractive targets to investigate mechanisms of initial fate choice, maturation of fate, and diversification of paths.

Overall, we also identified 20 different TFs with a requirement in cell-type formation or cell-type-specific transcription. Many of these FSTFs span the parenchymal and neural lineages, two of the more diverse tissue types of the animal. At least 40 different choices associated with distinct transcriptome states could be documented within the neoblasts. How neoblasts can choose between such a plethora of options is unknown. In the G_0_ state, 129 states were apparent, with transcriptomes displaying further maturation and distinction from one another. The timing of many choices in the trajectory of cells toward differentiation still remains unaccounted for and can be the subject of future investigation. Overall, this work helps define the scope, timing, and organization principles that define the generation of cell-type diversity in an adult context, in which tissue turnover and regeneration can produce all cells of the organism. We also present an atlas of TF signatures associated with stem cell differentiation into most cell types of a complete adult animal. This resource can enable probing of the mechanisms of cell-fate specification in regeneration and the study of the function and evolution of broadly conserved transcription factors in animal cell-type formation.

### Limitations of the study

It is possible that TFs with sparse or low expression in neoblasts or post-mitotic progenitors evaded detection in this atlas. For tissues where neoblast diversity was lower than post-mitotic progenitor diversity (e.g., neurons), it is possible that some fates were present and undetected because of lack of sufficient maturation of a specific transcriptome or because fate is harbored for that class in a different manner (e.g., epigenetically). The function of a number of TFs expressed in neoblast classes was assessed here. However, many TFs were unexplored and future work will be important in determining their roles, which could range from fate specification to controlling particular attributes of cell-type physiology.

## STAR★METHODS

### RESOURCE AVAILABILITY

#### Lead contact

Further information and requests for resources should be directed to and will be fulfilled by the Lead Contact, Peter W. Reddien (reddien@wi.mit.edu)

#### Materials availability

This study did not generate any unique reagents.

#### Data and code availability

- The 10X scRNA-seq dataset on X1 and X2 FACS-sorted cells generated here is available upon the date of publication at the Short Read Archive under the name “10X scRNA-seq of *Schmidtea*: X1 Neoblasts and G0 Progenitor Cells,” SRA: PRJNA1067154.
- This paper does not report original code
- Any additional information required to reanalyze the data reported in this paper is available from the lead contact upon request.

### EXPERIMENTAL MODEL AND STUDY PARTICIPANT DETAILS

Asexual *S. mediterranea* (strain CIW4) animals were maintained in 20°C incubators in 1X Montjuic salts (2mM NaCl, 0.1mM KCl, 0.1mM MgSO4, 0.12mM NaHCO3). Animals were starved for 1–2 weeks prior to experiments.

### METHOD DETAILS

#### Fluorescence-activated cell sorting (FACS)

Cell preparation for FACS was performed as described.^9^ Briefly: starved animals were diced in polystyrene dishes with Calcium-Magnesium Free solution (400 mg/L NaH2PO4, 800 mg/L NaCl, 1200 mg/L KCl, 800 mg/L NaHCO3, 240 mg/L glucose, 15mM HEPES, pH7.3) with 1%BSA and 0.1 mg/mL collagenase (termed CMFB-C solution). Diced fragments were transferred to a 50mL conical tube and agitated in 50mL of CMFB-C using transfer pipettes for 10 min to generate cell suspensions. The cell suspension was passed through a 40μm filter and centrifuged at 300g for 5 min. Supernatant was decanted and cells were resuspended in 3.5mL CMFB (no collagenase) before passing through a 35μm filter. 10 mg/mL Hoechst solution (Invitrogen) was added to cell suspensions at a 1:50 dilution and the suspension was incubated for 45 min in the dark at room temperature. Propidium iodide was added to cells (5 mg/mL) directly before sorting. Cells were sorted on a FACS Aria II (BD Biosciences) for live (propidium iodide negative), nucleated (Hoechst positive) cells.

#### Cell sorting, 10X libraries, and scRNA-seq

Cells were sorted into X1 and X2 gates as described.^35^ After sorting, cells were resuspended in CMF with 1% BSA in a concentration range between 700 cells/uL to 1200 cells/uL. Cells were processed using the 10X Genomics Chromium Controller and Chromium Single Cell Library and Gel Bread Kit following the standard manufacturer protocol. Amplified cDNA libraries were quantified using a bioanalyzer and size selected using AMPure beads. Sequencing was performed on an Illumina HighSeq 2500 using the ‘Drop-Seq core computation protocol’ as described in (Fincher et al., 2018). Reads were mapped to the v6 Dresden transcriptome assembly (Rozanski et al., 2019) and a gene cell matrix was generated for analysis using the Seurat 3.0.2 pipeline.

#### scRNA-seq analysis

The Seurat 3.0.2 package was used for pre-processing, quality control, clustering, and subclustering analyses.^64^ Each individual library was processed separately. First, contig isoforms for sequences were merged by summing the mapped reads to each isoform. Transcripts representing ribosomal and mitochondrial reads were removed as described,^1^ and cells with fewer than 500 UMIs or greater than 18,000 UMIs were also removed. Seurat objects were generated for each individual library. Cells were then normalized using the NormalizeData function (“LogNormalize” method; scale factor = 10,000) and variable genes were identified using the FindVariableGenes function (“vst” selection method; 2,000 features). Variations in global gene expression were regressed out by scaling and centering to the number of unique molecular identifiers (UMIs) using the ScaleData function. The same variable genes were used as input for principal component analysis using the RunPCA function. Cells were clustered using the FindNeighbors and FindClusters functions, and subsequently plotted in two dimensions using Uniform Manifold Approximation and Projection (UMAP; RunUMAP function). The number of principal components (PCs) used as input for FindClusters and RunUMAP was determined empirically by testing a range of different PCs as input until optimal clustering occurred, as described.^1^ The resolution parameter was set in a similar manner, to generate clusters that differ from each other in gene expression with their neighbors without generating clusters without differences in gene expression (‘overclustering’). Cells from individual libraries/objects and of the same FACS gating were merged using the merge function, whenever the same 10X Genomics Chromium Kit chemistry was used in library production. Upon generation, two clusters from the merged X1-gated object contained no exclusively enriched genes and the cells of the clusters had far lower UMI counts than other clusters, suggesting that these clusters represented artifacts (Fincher et al., 2018). These clusters were removed from the data. Additionally, two other clusters represented more differentiated populations of muscle and epidermis (clustering away from cycling muscle and epidermal neoblasts) – likely representing non-cycling cells – and were removed. Cells with expression of *smedwi-1* > 0.5ln (UMI-per-10,000 + 1) were retained for the final merged X1 dataset. X1 clusters were identified by the presence of known markers or novel markers associated with known differentiated tissues. For the merged G_0_ dataset, initial clustering yielded 26 clusters (PC = 55, resolution = 0.6). Finally, cells with expression of *smedwi-1* < 3.5ln (UMI-per-10,000 + 1) and *p4hb* > 1.5ln (UMI-per-10,000 + 1) were retained. G_0_ cell cluster labels were identified by the presence of known markers for differentiated tissues (Fincher et al., 2018). For both datasets (merged X1 and G_0_ progenitor cells), genes enriched in clusters and subclusters were identified using the FindMarkers functions (log-scaled fold difference threshold of 0.5).

When library production across multiple runs involved different 10X Genomics Chromium Kit chemistries (e.g., Chromium v3 vs. v3.1), the Seurat Integration pipeline was used rather than the merge function alone. Briefly, objects were first merged (merge function) then split into lists of two objects (SplitObject function). Cells were normalized, variable genes identified, and features repeatedly variable across datasets were selected (SelectIntegrationFeatures function). Cross-data pairs of cells in a matched biological state (called anchors) were identified (FindIntegrationAnchors function) and an integrated data assay was formed (IntegrateData function). The default assay was then set to “integrated”, and standard scaling, PCA, UMAP, and clustering were performed. The default assay was switched to “RNA” when visualizing gene expression and switched back to “integrated” when subclustering. Subclustering analysis was performed by subsetting data into new Seurat objects (subset function) and performing processing similar to initial cluster formation (variable features, scaling data, PCA, finding neighbors and clusters, UMAP).

#### PaCMAP visualization and Leiden clustering

PaCMAP dimensionality reduction and visualization was performed by importing top principal components, calculated in Seurat, into python for use with the PaCMAP package.^66^ PaCMAP positions of each cell were then imported back into R and visualized according to original Louvain cluster identities for comparison to UMAP-based visualization.

Leiden clustering was performed in Seurat using the same number of principal components used during Louvain clustering. The resolutions were set to produce a similar number of clusters as Louvain clustering. Adjusted Rand Indices were calculated between cluster identities generated by k-means, Louvain, Leiden clustering, and random label shuffling using different random seeds.

#### URD

Transcriptional pseudotime trajectory analysis was performed in R using the URD package.^69^ Specified neoblasts and post-mitotic progenitors were combined for muscle and neural specified cells separately. Genes used for analysis include the intersection of variable genes across specialized neoblasts and post-mitotic progenitors, the intersection of enriched marker genes from neoblast and post-mitotic progenitor clustering, and *smedwi-1* and *p4hb*. This produced 88 genes used for neural-specified cells and 73 genes used for muscle-specified cells. Neural subcluster 3 and muscle subcluster 0 were used as the roots of the trajectories. Diffusion maps were generated with knn = 90 and sigma = 5. Louvain clustering of G0 cells to set terminal tip identities was done with num.nn = 50. Pseudotime logistics were determined using optimal.cells.forward = 30 and max.cells.back = 50. Random walks were simulated 100,000 times. Trees were constructed using divergence.method = “preference”, cells.per.pseudotime.bin = 30, bins.per.pseudotime.window = 5, p.thresh = 0.005. Cells along terminal segment trajectories were identified and those cells were plotted in Seurat for visualization.

#### RNAi

RNAi was performed as described in. Briefly, dsRNA was synthesized by *in vitro* transcription (Promega) using PCR-generated templates with flanking T7 promoters, followed by ethanol precipitation, and annealing after suspension in water. dsRNA was mixed with planarian food (liver) and ~2 μL of this mixture was given per animal for feedings. All animals received at least 8 feedings. For regeneration experiments, animals were amputated intro three pieces (head, trunk, and tail) several days after the last RNAi feeding. Fragments were fixed for labeling and further analysis. RNAi experiments were repeated at least once to show reproducibility.

#### Cell fluorescence *in situ* hybridization

This protocol was performed as described.^9^ Briefly, sorted cells were resuspended at 4,000 cells/μL in CMF and plated on polylysine-D coverslips in 24 well plates. Cells were allowed to settle for 30 min before fixation in 4% paraformaldehyde in CMF. Cells were washed in 1% phosphate-buffered saline with 0.1% Triton X-(PBSTx) and a 1:1 PBSTx:PreHyb solution (King and Newmark, 2013). Cells were incubated in PreHyb for 2 h at 56°C and hybridized with RNA probes at 1:800 in Hyb for >16 h at 56°C (digoxigenin-, fluorescein isothiocynate-, and dintriophenol-labeled riborpobes were synthesized as described in Pearson et al. 2009). Cells were then blocked 30 min prior to incubating overnight with anti-DIG-POD (1:1500, Roche), anti-FITC-POD (1:2000, Roche), or anti-DNP-HRP (1:150 PerkinElmer) in blocking solutions of PBSTx with 10% western block reagent (Roche) for ant-DIG-POD, combined 5% western block reagent and 5% casein solution (Sigma) for anti-FITC-POD, or combined 5% western block reagent and 5% heat-in-activated horse serum anti-DNP-HRP. Fluorescent tyramide signal amplification involved cells placed in borate buffer for 5 min (0.1M boric acid, 2M NaCl, pH8.5), followed by 10 min in borate buffer with rhodamine (1:1000) or fluorescein (1:1500) tyramide, and 0.0003% hydrogen peroxide. Peroxidase inactivation was then performed in 1% sodium azide for >1 h. For double FISH, this was followed by antibody labeling for the second probe. Samples were mounted in Vectashield with DAPI (Vector Labs).

#### Fixation and *in situ* hybridization

Animals were incubated in 5% N-acetylcysteine for 5 min prior to fixation with 4% formaldehyde in PBSTx for 20 min (with a brief PBSTx wash in between). Animals were then washed in PBSTx once, placed in PBSTx:Methanol for 10 min, and methanol alone for 10 min before storage at —20C overnight. Animals were then bleached for 2 h in 5% formamide, 0.5x SSC, and 1.2% hydrogen peroxide solution on a light source. Animals were then incubated in 5 μg/mL proteinase K, before post-fixation in 4% formaldehyde in PBSTx. Hybridization and tyramide development was performed as described above.

#### Imaging acquisition

Fluorescent micrographs were acquired using the Leica Stellaris Sp8 laser scanning confocal microscope. ImageJ software (Fiji) was used for processing images from FISH data.

#### Transcription factor catalog construction

A FASTA database of planarian protein sequences was generated from six-frame translations of all dd_Smed_v6 transcripts.^63^ Pfam Hidden Markov models (HMMs) were downloaded from pfam.xfam.org for known DNA-binding domains (DBD) and collated to a single file.^68^ Using HMMER v3, the translated transcriptome was searched (hmmsearch) using each DBD HHM (hmmfetch) to identify transcripts encoding possible transcription factors (Table S2; raw output). Of transcripts representing multiple isoforms of the same gene, only the longest transcript containing a DBD was recorded. Planarian transcripts with at least one match to a DBD HHM were compiled into an intermediate catalog and were BLAST searched (blastx; evalue < 1e-5) against the human proteome. Genes that have been previously identified in planarians were identified by constructing a blast database from *Schmidtea mediterranea* proteins and transcripts previously deposited into GenBank, and BLAST searching against that (blastx and blastn; evalue < 1e-5, percent identity >90%). Probable transcription factors were identified by homology to human transcription factors and the subset of validated FSTFs were identified by literature searching for planarian transcription factors previously deposited in GenBank. Transcripts that blast to non-transcription factors were categorized as non-transcription factor DNA-related proteins or miscellaneous proteins and were excluded. Transcript products without similarity to a human protein were classified as having no blast hit and were retained for future analysis.

#### Transcription factor correlation analysis

Gene-cell matrices of sequenced X1 and G_0_ progenitor cells were exported from Seurat after preprocessing and clustering as csv files of the gene-cell matrix, cell identities, and transcript contig identifiers and imported to MATLAB. Putative transcription factors from the catalog were isolated from both gene cell matrices, collapsing all isoforms for each gene. Pearson correlation coefficients (corrcoef) were calculated between gene pairs across X1 or G_0_ cells. The technique was validated by making a correlation matrix of known FSTFs from the transcription factor catalog and verifying high correlations between transcription factors with overlapping expression. Putative transcription factors from the catalog with high correlations (r > 0.14) between either each other or a known FSTF were inspected. Heatmaps were generated comparing correlations between correlated putative transcription factors and between putative transcription factors and known FSTFs. Genes in heatmaps were ordered by hierarchical clustering across both axes. Correlation matrices were exported to Python as csv files for figure generation using the Seaborn library. Final correlation matrices were reclustered by Euclidian distance using the UPGMA algorithm, and gene pairs with the highest correlations were included in the final figure.

#### Transcription factor modules

Transcription factor module figures were generated by extracting UMAP positions of cells expressing the entire module of transcription factors (>0.5ln (UMI-per-10,000 + 1)). The cell positions were binned to a 2D heatmap and contours were generated by convolving a Gaussian kernel over it (with a standard deviation of 2*the minimal dimension of the UMAP plot) and thresholding (0.5–0.7 of the maximal value).

#### Ratkowsky index

Cell principal component embeddings were extracted for specific clusters in Seurat. The number of principal components chosen for each tissue type was determined by the elbow method, corresponding to the number of principal components where additional components no longer explain more variance than what is expected by splitting unstructured data. The same number of principal components were used for S/G_2_/M neoblast clusters and for G_0_ progenitor clusters (Neural: 40, Muscle: 20, Cathepsin: 20). Ratkowsky indicies were calculated using the NbClust package in R for these principal component embeddings using kmeans clustering and euclidean distance.^67^ NbClust function source code was altered to remove hard-coded seed state and replaced with a random number generator and this was run 1,000 times per tissue type to generate an average Ratkowsky index and a 95% confidence interval. The maximum Ratkowsky value is indicated on Ratkowsky index plots.

#### Transcription factor enrichment figures

Transcription factor tissue-type expression plots for S/G_2_/M neoblasts were made by using the ‘FindMarkers’ command with pseudocount = 0 for identities set to all clusters corresponding to the same major tissue type. These were then filtered for transcription factors and binned by their DNA-binding domain. Transcription factor with enriched expression (p value <0.05) were plotted according to their log 2-fold change enrichment (log2FC). The homeodomain-containing transcription factor heatmap was generated by averaging expression for homeodomain-containing genes across each tissue type, groupedas before. A ratio of average expression for each tissue divided by max average expression was generated for each transcription factor. The heatmap was plotted using the seaborn package in python^65^ and hierarchically clustered.

#### Transcription factor atlas

The transcription factor atlas table for G_0_ progenitor cells was generated by creating a Seurat object of all G_0_ cells where the cluster identity of each cell was determined by the subclustering of the clusters from the tissue-specific analysis. Enriched genes for each subcluster were then found in Seurat using the ‘FindAllMarkers’ command implementing the MAST statistical test with pseudocount = 0 and filtered for transcription factors with at least a 1.5 log2FC enrichment, an adjusted p value of 0.001, and have expression >0 in at least 20% of cells within the subcluster they are enriched in. Transcription factors were sorted by increasing adjusted p value and the top 8 were included in the transcription factor atlas.

#### Neural G_0_ characterization

Neural G_0_ clusters were individually subclustered, with neighboring clusters 12 and 15 subclustered together. Enriched genes were identified for each subcluster in the same manner as other tissue types. To relate G_0_ subclusters to their corresponding subcluster in the Fincher et al. 2018 atlas of differentiated cells, we created a merged Seurat object of all G_0_ neural cells, with each cell maintaining their identity from subclustering. We generated a similar merged Seurat object for non-ciliated, ciliated, and “All Neural” clusters 3, 4, 7, 8, 10, 36, 43, and 59 (clusters that were not annotated as ciliated or non-ciliated, and were not enriched in expression of the neoblast genes *smedwi-1* or *smedwi-2*) from the Fincher et al. 2018 dataset.

From these merged Seurat datasets, we found genes commonly enriched in at least one subcluster in both datasets and calculated the Euclidian distance between all G_0_ and differentiated/Fincher et al. 2018 subclusters based on the average z-scored expression of just these mutually-enriched genes. For G_0_ and differentiated pairs with low distances, we looked for the expression of unique marker genes shared between these cells to indicate a possible shared lineage. We annotated these predicted corresponding differentiated subclusters on UMAP plots of each neural G_0_ subclustering. We also annotated neural G_0_ UMAP plots with genes enriched in each subcluster.

#### Gene annotation and nomenclature

Genes were labeled as previously described.^1^ Briefly, previously published planarian genes that had been submitted to the NCBI Nucleotide database (https://www.ncbi.nlm.nih.gov/nucleotide/) appear in *italics* within text and figures. Sequences not found in the planarian database, but that have a human best-blast (blastx) are labeled in uppercase with their human gene name, followed by the contig ID of the dd_v6 transcriptome assembly in parentheses.

### QUANTIFICATION AND STATISTICAL ANALYSIS

Description for all quantification and statistical analysis can be found in the appropriate Method Details section. Numbers of animals that appear like the given control animal or have a phenotype like the given RNAi animal are given in the supplemental figures under the gene name within each FISH image. These were scored blindly. Transcription factor atlas (Table S6) genes have corresponding adjusted p values in ‘p value’ sheets within the file. Statistical programs used include R and Python, and details of packages are listed in appropriate method details section and in the key resources table.

## Supplementary Material

1

2

3

4

5

6

7

## Figures and Tables

**Table:** KEY RESOURCES TABLE

REAGENT or RESOURCE	SOURCE	IDENTIFIER

Antibodies		

anti-Digoxigenin-POD	Roche	Cat# 11 207 733 910; RRID: AB_514500
anti-Fluorescein-POD	Roche	Cat# 11 426 346 910; RRID: AB_840257
anti-DNP-HRP	Perkin-Elmer	Cat# FP1129; RRID: AB_2629439

Chemicals, peptides, and recombinant proteins		

Hoechst	Invitrogen	Cat# H3570
BSA	Sigma Aldrich	Cat #A8806
Western Blocking Reagent	Roche Diagnostics	Cat# WESTBL-RO
Casein	Sigma Aldrich	Cat# B6429
Vectashield with DAPI	Vector Laboratories	Cat# H-1200
N-acetylcysteine	Sigma Aldrich	Cat #A7250
Formaldehyde	Fisher Scientific	Cat# F79500
Collagenase	Sigma Aldrich	Cat #C0130–500MG
Propridium iodide (PI)	Sigma Aldrich	Cat# P4864
T7 RNA Polymerase	Promega	Cat# P207B

Deposited data		

DropSeq planarian atlas	Fincher et al. 2018^1^	GEO series accession GSE111764
Dresden transcriptome v6	Rozanski et al. 2019^63^	http://planmine.mpi-cbg.de/planmine/begin.do
10X scRNA-seq: X1 and G0 Cells	This Paper	SRA: PRJNA1067154

Experimental models: Organisms/strains		

Asexual Schmidtea mediterranea strain CIW4	laboratory strain	N/A

Recombinant DNA		

pGEM vector	Promega	Cat# A1360

Software and algorithms		

Seurat R Package	Satijaetal., 2015^64^	https://satijalab.org/seurat
Drop-seq core computational protocol v1.2	James Nemesh and Steve McCarroll	https://github.com/broadinstitute/Drop-seq/releases
FIJI	ImageJ	https://imagej.net/Fiji
ZEN digital imaging software	Zeiss	https://www.zeiss.com/microscopy/us/products/microscope-software/zen.html
Seaborn Python Package	Waskom, M.L., 2021^65^	seaborn.pydata.org
PaCMAP Python Package	Wangetal., 2021^66^	pypi.org/project/pacmap/
NbClust R Package	Charradetal. 2014^67^	N/A
HHMER v3	Sean R. Eddy	hmmer.org
Pfam	J. Mistryetal. 2021^68^	pfam.xfam.org
URD	Farrell etal. 2018^69^	github.com/farrellja/URD

## Highlights

- Systematic identification of stem cell and post-mitotic progenitor states
- Transcription factor expression signatures for all detected fate choices
- RNAi-based establishment of transcription factor roles in progenitor fate
- Diversification of fate at stem cell or post-mitotic progenitor stages

## References

[1] Fincher CT, Wurtzel O, de Hoog T, Kravarik KM, and Reddien PW (2018). Cell type transcriptome atlas for the planarian *Schmidtea mediterranea*. Science 360, eaaq1736. 10.1126/science.aaq1736. PMC6563842 29674431

[2] Baguñà J, Saló E, and Auladell C. (1989). Regeneration and pattern formation in planarians. III. Evidence that neoblasts are totipotent stem cells and the source of blastema cells. Development 107, 77–86.

[3] Wagner DE, Wang IE, and Reddien PW (2011). Clonogenic neoblasts are pluripotent adult stem cells that underlie planarian regeneration. Science 332, 811–816. 10.1126/science.1203983. 21566185 PMC3338249

[4] Sulston JE, Schierenberg E, White JG, and Thomson JN (1983). The embryonic cell lineage of the nematode Caenorhabditis elegans. Dev. Biol 100, 64–119. 10.1016/0012-1606(83)90201-4. 6684600

[5] Reddien PW (2018). The Cellular and Molecular Basis for Planarian Regeneration. Cell 175, 327–345. 10.1016/j.cell.2018.09.021. 30290140 PMC7706840

[6] King RS, and Newmark PA (2012). The cell biology of regeneration. J. Cell Biol 196, 553–562. 10.1083/jcb.201105099. 22391035 PMC3307701

[7] Newmark PA, and Sá nchez Alvarado A. (2000). Bromodeoxyuridine specifically labels the regenerative stem cells of planarians. Dev. Biol 220, 142–153. 10753506 10.1006/dbio.2000.9645

[8] Wenemoser D, and Reddien PW (2010). Planarian regeneration involves distinct stem cell responses to wounds and tissue absence. Dev. Biol 344, 979–991, S0012–1606(10)00837–7 [pii]. 10.1016/j.ydbio.2010.06.017. 20599901 PMC2950745

[9] Raz AA, Wurtzel O, and Reddien PW (2021). Planarian stem cells specify fate yet retain potency during the cell cycle. Cell Stem Cell 28, 1307–1322.e5. 10.1016/j.stem.2021.03.021. 33882291 PMC8254784

[10] Adler CE, Seidel CW, McKinney SA, and Sánchez Alvarado A. (2014). Selective amputation of the pharynx identifies a *FoxA*-dependent regeneration program in planaria. Elife 3, e02238. 10.7554/eLife.02238. PMC3985184 24737865

[11] Cowles MW, Brown DDR, Nisperos SV, Stanley BN, Pearson BJ, and Zayas RM (2013). Genome-wide analysis of the bHLH gene family in planarians identifies factors required for adult neurogenesis and neuronal regeneration. Development 140, 4691–4702. 10.1242/dev.098616. 24173799

[12] Cowles MW, Omuro KC, Stanley BN, Quintanilla CG, and Zayas RM (2014). COE loss-of-function analysis reveals a genetic program underlying maintenance and regeneration of the nervous system in planarians. PLoS Genet. 10, e1004746. 10.1371/journal.pgen.1004746. PMC4214590 25356635

[13] Currie KW, and Pearson BJ (2013). Transcription factors *lhx1/5–1* and *pitx* are required for the maintenance and regeneration of serotonergic neurons in planarians. Development 140, 3577–3588. 10.1242/dev.098590. 23903188

[14] He X, Lindsay-Mosher N, Li Y, Molinaro AM, Pellettieri J, and Pearson BJ (2017). FOX and ETS family transcription factors regulate the pigment cell lineage in planarians. Development 144, 4540–4551. 29158443 10.1242/dev.156349 PMC5769623

[15] Lapan SW, and Reddien PW (2011). *dlx* and *sp6–9* control optic cup regeneration in a prototypic eye. PLoS Genet. 7, e1002226, PGENETICS-D-11–00610 [pii]. 10.1371/journal.pgen.1002226. PMC3154955 21852957

[16] Lapan SW, and Reddien PW (2012). Transcriptome Analysis of the Planarian Eye Identifies *ovo* as a Specific Regulator of Eye Regeneration. Cell Rep. 2, 294–307. 10.1016/j.celrep.2012.06.018. 22884275 PMC3785364

[17] Mä rz M, Seebeck F, and Bartscherer K. (2013). A Pitx transcription factor controls the establishment and maintenance of the serotonergic lineage in planarians. Development 140, 4499–4509. 10.1242/dev.100081. 24131630

[18] Molinaro AM, and Pearson BJ (2016). In silico lineage tracing through single cell transcriptomics identifies a neural stem cell population in planarians. Genome Biol. 17, 87. 10.1186/s13059-016-0937-9. 27150006 PMC4858873

[19] Roberts-Galbraith RH, Brubacher JL, and Newmark PA (2016). A functional genomics screen in planarians reveals regulators of whole-brain regeneration. Elife 5, e17002. 10.7554/eLife.17002. PMC5055394 27612384

[20] Ross KG, Currie KW, Pearson BJ, and Zayas RM (2017). Nervous system development and regeneration in freshwater planarians. Wiley interdisciplinary reviews. Developmental biology 6. 10.1002/wdev.266. 28326682

[21] Ross KG, Molinaro AM, Romero C, Dockter B, Cable KL, Gonzalez K, Zhang S, Collins EMS, Pearson BJ, and Zayas RM (2018). SoxB1 activity regulates sensory neuron regeneration, maintenance, and function in planarians. Dev. Cell 47, 331–347.e5. 10.1016/j.devcel.2018.10.014. 30399335

[22] Scimone ML, Srivastava M, Bell GW, and Reddien PW (2011). A regulatory program for excretory system regeneration in planarians. Development 138, 4387–4398, 138/20/4387 [pii]. 10.1242/dev.068098. 21937596 PMC3177309

[23] Scimone ML, Kravarik KM, Lapan SW, and Reddien PW (2014). Neoblast Specialization in Regeneration of the Planarian *Schmidtea mediterranea*. Stem Cell Rep. 3, 339–352. 10.1016/j.stemcr.2014.06.001. PMC4176530 25254346

[24] Scimone ML, Lapan SW, and Reddien PW (2014). A *forkhead* transcription factor is wound-induced at the planarian midline and required for anterior pole regeneration. PLoS Genet. 10, e1003999. 10.1371/journal.pgen.1003999. PMC3886891 24415944

[25] Scimone ML, Cote LE, and Reddien PW (2017). Orthogonal muscle fibres have different instructive roles in planarian regeneration. Nature 551, 623–628. 10.1038/nature24660. 29168507 PMC6263039

[26] Scimone ML, Wurtzel O, Malecek K, Fincher CT, Oderberg IM, Kravarik KM, and Reddien PW (2018). foxF-1 Controls Specification of Non-body Wall Muscle and Phagocytic Cells in Planarians. Curr. Biol 28, 3787–3801.e6. 10.1016/j.cub.2018.10.030. 30471994 PMC6411049

[27] van Wolfswinkel JC, Wagner DE, and Reddien PW (2014). Single-Cell Analysis Reveals Functionally Distinct Classes within the Planarian Stem Cell Compartment. Cell Stem Cell 15, 326–339. 10.1016/j.stem.2014.06.007. 25017721 PMC4171737

[28] Vásquez-Doorman C, and Petersen CP (2014). *zic-1* Expression in planarian neoblasts after injury controls anterior pole regeneration. PLoS Genet. 10, e1004452. 10.1371/journal.pgen.1004452. PMC4081000 24992682

[29] Vogg MC, Owlarn S, Pé rez Rico YA, Xie J, Suzuki Y, Gentile L, Wu W, and Bartscherer K. (2014). Stem cell-dependent formation of a functional anterior regeneration pole in planarians requires Zic and Forkhead transcription factors. Dev. Biol 390, 136–148. 10.1016/j.ydbio.2014.03.016. 24704339

[30] Wang C, Han XS, Li FF, Huang S, Qin YW, Zhao XX, and Jing Q. (2016). Forkhead containing transcription factor Albino controls tetrapyrrole-based body pigmentation in planarian. Cell Discov. 2, 16029. 10.1038/celldisc.2016.29. PMC4969599 27551436

[31] Wenemoser D, Lapan SW, Wilkinson AW, Bell GW, and Reddien PW (2012). A molecular wound response program associated with regeneration initiation in planarians. Genes Dev. 26, 988–1002. 10.1101/gad.187377.112. 22549959 PMC3347795

[32] Zeng A, Li H, Guo L, Gao X, McKinney S, Wang Y, Yu Z, Park J, Semerad C, Ross E, (2018). Prospectively Isolated Tetraspanin(+) Neoblasts Are Adult Pluripotent Stem Cells Underlying Planaria Regeneration. Cell 173, 1593–1608.e20. 10.1016/j.cell.2018.05.006. 29906446 PMC9359418

[33] Plass M, Solana J, Wolf FA, Ayoub S, Misios A, Glažar P, Obermayer B, Theis FJ, Kocks C, and Rajewsky N. (2018). Cell type atlas and lineage tree of a whole complex animal by single-cell transcriptomics. Science 360, eaaq1723. 10.1126/science.aaq1723. 29674432

[34] Niu K, Xu H, Xiong YZ, Zhao Y, Gao C, Seidel CW, Pan X, Ying Y, and Lei K. (2021). Canonical and early lineage-specific stem cell types identified in planarian SirNeoblasts. Cell Regen. 10, 15. 10.1186/s13619-021-00076-6. 33740162 PMC7979843

[35] Hayashi T, Asami M, Higuchi S, Shibata N, and Agata K. (2006). Isolation of planarian X-ray-sensitive stem cells by fluorescence-activated cell sorting. Dev. Growth Differ 48, 371–380. 16872450 10.1111/j.1440-169X.2006.00876.x

[36] Reddien PW (2022). Positional Information and Stem Cells Combine to Result in Planarian Regeneration. Cold Spring Harbor Perspect. Biol 14, a040717. 10.1101/cshperspect.a040717. PMC9121904 34518341

[37] Neiro J, Sridhar D, Dattani A, and Aboobaker A. (2022). Identification of putative enhancer-like elements predicts regulatory networks active in planarian adult stem cells. Elife 11, e79675. 10.7554/eLife.79675. PMC9522251 35997250

[38] Eddy SR (2011). Accelerated Profile HMM Searches. PLoS Comput. Biol 7, e1002195. 10.1371/journal.pcbi.1002195. PMC3197634 22039361

[39] Wheeler TJ, and Eddy SR (2013). nhmmer: DNA homology search with profile HMMs. Bioinformatics 29, 2487–2489. 10.1093/bioinformatics/btt403. 23842809 PMC3777106

[40] Reilly MB, Cros C, Varol E, Yemini E, and Hobert O. (2020). Unique homeobox codes delineate all the neuron classes of C. elegans. Nature 584, 595–601. 10.1038/s41586-020-2618-9. 32814896 PMC7587405

[41] Cebrià F. (2016). Planarian Body-Wall Muscle: Regeneration and Function beyond a Simple Skeletal Support. Front. Cell Dev. Biol 4, 8. 10.3389/fcell.2016.00008. 26904543 PMC4744845

[42] Cote LE, Simental E, and Reddien PW (2019). Muscle functions as a connective tissue and source of extracellular matrix in planarians. Nat. Commun 10, 1592. 10.1038/s41467-019-09539-6. 30962434 PMC6453901

[43] Scimone ML, Atabay KD, Fincher CT, Bonneau AR, Li DJ, and Reddien PW (2020). Muscle and neuronal guidepost-like cells facilitate planarian visual system regeneration. Science 368, eaba3203. 10.1126/science.aba3203. PMC8128157 32586989

[44] Scimone ML, Cote LE, Rogers T, and Reddien PW (2016). Two FGFRL-Wnt circuits organize the planarian anteroposterior axis. Elife 5, e12845. 10.7554/eLife.12845. PMC4865367 27063937

[45] Witchley JN, Mayer M, Wagner DE, Owen JH, and Reddien PW (2013). Muscle cells provide instructions for planarian regeneration. Cell Rep. 4, 633–641. 10.1016/j.celrep.2013.07.022. 23954785 PMC4101538

[46] Hyman LH (1951). The Invertebrates: Platyhelminthes and Rhynchocoela the Acoelomate Bilateria (McGraw-Hill Book Company Inc.).

[47] Chong T, Collins JJ 3rd, Brubacher JL, Zarkower D, and Newmark PA (2013). A sex-specific transcription factor controls male identity in a simultaneous hermaphrodite. Nat. Commun 4, 1814. 10.1038/ncomms2811. 23652002 PMC3674237

[48] Brown DDR, Molinaro AM, and Pearson BJ (2018). The planarian TCF/LEF factor Smed-tcf1 is required for the regeneration of dorsal-lateral neuronal subtypes. Dev. Biol 433, 374–383. 10.1016/j.ydbio.2017.08.024. 29291981

[49] Issigonis M, Redkar AB, Rozario T, Khan UW, Mejia-Sanchez R, Lapan SW, Reddien PW, and Newmark PA (2022). A Kruppel-like factor is required for development and regeneration of germline and yolk cells from somatic stem cells in planarians. PLoS Biol. 20, e3001472. 10.1371/journal.pbio.3001472. PMC9286257 35839223

[50] Khan UW, and Newmark PA (2022). Somatic regulation of female germ cell regeneration and development in planarians. Cell Rep. 38, 110525. 10.1016/j.celrep.2022.110525. PMC8994625 35294875

[51] Saberi A, Jamal A, Beets I, Schoofs L, and Newmark PA (2016). GPCRs Direct Germline Development and Somatic Gonad Function in Planarians. PLoS Biol. 14, e1002457. 10.1371/journal.pbio.1002457. PMC4862687 27163480

[52] Wang Y, Zayas RM, Guo T, and Newmark PA (2007). *nanos* function is essential for development and regeneration of planarian germ cells. Proc. Natl. Acad. Sci. USA 104, 5901–5906. 17376870 10.1073/pnas.0609708104 PMC1851589

[53] Forsthoefel DJ, Cejda NI, Khan UW, and Newmark PA (2020). Cell-type diversity and regionalized gene expression in the planarian intestine. Elife 9, e52613. 10.7554/eLife.52613. PMC7117911 32240093

[54] Flores NM, Oviedo NJ, and Sage J. (2016). Essential role for the planarian intestinal GATA transcription factor in stem cells and regeneration. Dev. Biol 418, 179–188. 10.1016/j.ydbio.2016.08.015. 27542689 PMC5055475

[55] González-Sastre A, De Sousa N, Adell T, and Saló, E. (2017). The pioneer factor *Smed-gata456–1* is required for gut cell differentiation and maintenance in planarians. Int. J. Dev. Biol 61, 53–63. 10.1387/ijdb.160321es. 28287248

[56] Eisenhoffer GT, Kang H, and Sá nchez Alvarado, A. (2008). Molecular analysis of stem cells and their descendants during cell turnover and regeneration in the planarian *Schmidtea mediterranea*. Cell Stem Cell 3, 327–339. 18786419 10.1016/j.stem.2008.07.002 PMC2614339

[57] Wurtzel O, Oderberg IM, and Reddien PW (2017). Planarian Epidermal Stem Cells Respond to Positional Cues to Promote Cell-Type Diversity. Dev. Cell 40, 491–504.e5. 10.1016/j.devcel.2017.02.008. 28292427 PMC5679284

[58] Tu KC, Cheng LC, T K Vu H, Lange JJ, McKinney SA, Seidel CW, and Sánchez Alvarado A. (2015). *Egr-5* is a post-mitotic regulator of planarian epidermal differentiation. Elife 4, e10501. 10.7554/eLife.10501. PMC4716842 26457503

[59] Wagner DE, Ho JJ, and Reddien PW (2012). Genetic regulators of a pluripotent adult stem cell system in planarians identified by RNAi and clonal analysis. Cell Stem Cell 10, 299–311. 22385657 10.1016/j.stem.2012.01.016 PMC3338251

[60] Rink JC, Vu HTK, and Sánchez Alvarado A. (2011). The maintenance and regeneration of the planarian excretory system are regulated by EGFR signaling. Development 138, 3769–3780. 10.1242/dev.066852. 21828097 PMC3152929

[61] Vu HT, Rink JC, McKinney SA, McClain M, Lakshmanaperumal N, Alexander R, and Sá nchez Alvarado A. (2015). Stem cells and fluid flow drive cyst formation in an invertebrate excretory organ. Elife 4. 10.7554/eLife.07405. PMC4500094 26057828

[62] Park C, Owusu-Boaitey KE, Valdes GM, and Reddien PW (2023). Fate specification is spatially intermingled across planarian stem cells. Nat. Commun 14, 7422. 10.1038/s41467-023-43267-2. 37973979 PMC10654723

[63] Rozanski A, Moon H, Brandl H, Martín-Durán JM, Grohme MA, Hüttner K, Bartscherer K, Henry I, and Rink JC (2019). PlanMine 3.0-improvements to a mineable resource of flatworm biology and biodiversity. Nucleic Acids Res. 47, D812–D820. 10.1093/nar/gky1070. 30496475 PMC6324014

[64] Satija R, Farrell JA, Gennert D, Schier AF, and Regev A. (2015). Spatial reconstruction of single-cell gene expression data. Nat. Biotechnol 33, 495–502. 10.1038/nbt.3192. 25867923 PMC4430369

[65] Waskom M. (2021). seaborn: statistical data visualization. J. Open Source Softw 6, 3021. 10.21105/joss.03021.

[66] Wang Y, Huang H, Rudin C, and Shaposhnik Y. (2021). Understanding How Dimension Reduction Tools Work: An Empirical Approach to Deciphering t-SNE, UMAP, TriMap, and PaCMAP for Data Visualization. J. Mach. Learn. Res 22, 9129–9201.

[67] Charrad M, Ghazzali N, Boiteau V, and Niknafs A. (2014). NbClust: An R Package for Determining the Relevant Number of Clusters in a Data Set. J. Stat. Software 61, 1–36. 10.18637/jss.v061.i06.

[68] Mistry J, Chuguransky S, Williams L, Qureshi M, Salazar GA, Sonnhammer ELL, Tosatto SCE, Paladin L, Raj S, Richardson LJ, (2021). Pfam: The protein families database in 2021. Nucleic Acids Res. 49, D412–D419. 10.1093/nar/gkaa913. 33125078 PMC7779014

[69] Farrell JA, Wang Y, Riesenfeld SJ, Shekhar K, Regev A, and Schier AF (2018). Single-cell reconstruction of developmental trajectories during zebrafish embryogenesis. Science 360, eaar3131. 10.1126/science.aar3131. PMC6247916 29700225

