# Supplementary material for: A transcription factor atlas of stem cell fate in planarians
Source: Cell Rep. Author manuscript; Available in PMC 2024 Jul 9. (PMC11232438; doi:10.1016/j.celrep.2024.113843)
Supplement: 1 [file NIHMS1980910-supplement-1.pdf]

Cell Reports, Volume *43*

Supplemental information

A transcription factor

atlas of stem cell fate in planarians

Hunter O. King, Kwadwo E. Owusu-Boaitey, Christopher T. Fincher, and Peter W. Reddien

Figure S1

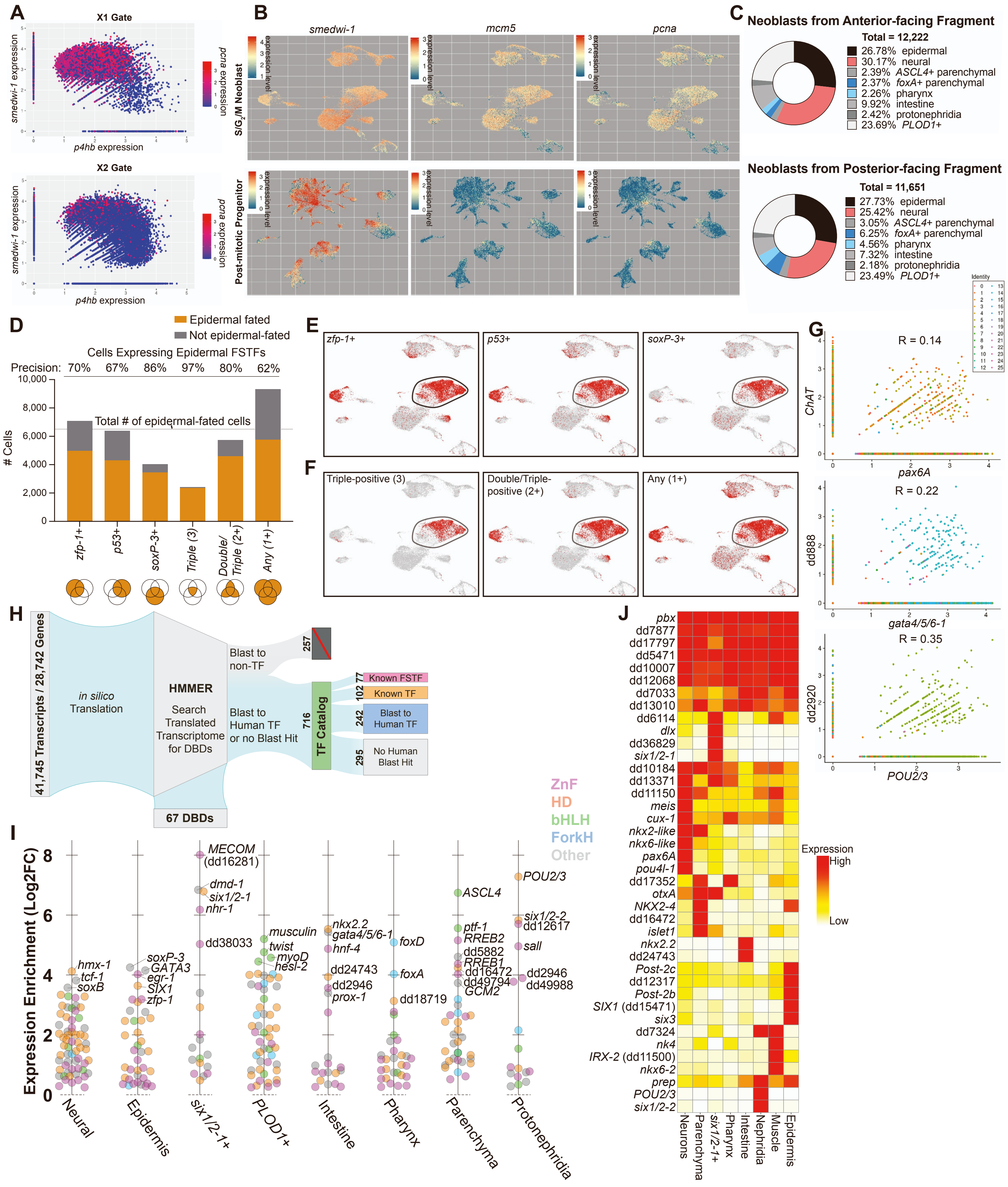
A
X1 Gate
smedwi-1 expression
p4hb expression
pcna expression
X2 Gate
B
smedwi-1
mcm5
pcna
S/G2/M Neoblast
Post-mitotic Progenitor
expression level
C
Neoblasts from Anterior-facing Fragment
Total = 12,222
26.78% epidermal
30.17% neural
2.39% ASCL4+ parenchymal
2.37% foxA+ parenchymal
2.26% pharynx
9.92% intestine
2.42% protonephridia
23.69% PLOD1+
Neoblasts from Posterior-facing Fragment
Total = 11,651
27.73% epidermal
25.42% neural
3.05% ASCL4+ parenchymal
6.25% foxA+ parenchymal
4.56% pharynx
7.32% intestine
2.18% protonephridia
23.49% PLOD1+
D
Epidermal fated
Not epidermal-fated
Cells Expressing Epidermal FSTFs
Precision: 70% 67% 86% 97% 80% 62%
Cells
Total # of epidermal-fated cells
zfp-1+
p53+
soxP-3+
Triple (3)
Double/ Triple (2+)
Any (1+)
E
zfp-1+
p53+
soxP-3+
F
Triple-positive (3)
Double/Triple-positive (2+)
Any (1+)
G
Identity
R = 0.14
ChAT
pax6A
R = 0.22
dd888
gata4/5/6-1
R = 0.35
dd2920
POU2/3
H
41,745 Transcripts / 28,742 Genes
in silico Translation
HMMER
Search Translated Transcriptome for DBDs
67 DBDs
Blast to non-TF
257
Blast to Human TF or no Blast Hit
716
TF Catalog
77 Known FSTF
102 Known TF
242 Blast to Human TF
295 No Human Blast Hit
I
Expression Enrichment (Log2FC)
ZnF
HD
bHLH
ForkH
Other
hmx-1
tcf-1
soxB
soxP-3
GATA3
egr-1
SIX1
zfp-1
MECOM (dd16281)
dmd-1
six1/2-1
nhr-1
dd38033
musculin
twist
myoD
hesl-2
nkx2.2
gata4/5/6-1
hnf-4
dd24743
dd2946
prox-1
foxD
foxA
dd18719
ASCL4
ptf-1
RREB2
dd5882
RREB1
dd16472
dd49794
GCM2
POU2/3
six1/2-2
dd12617
sall
dd2946
dd49988
Neural
Epidermis
six1/2-1+
PLOD1+
Intestine
Pharynx
Parenchyma
Protonephridia
J
pbx
dd7877
dd17797
dd5471
dd10007
dd12068
dd7033
dd13010
dd6114
dlx
dd36829
six1/2-1
dd10184
dd13371
dd11150
meis
cux-1
nkx2-like
nkx6-like
pax6A
pou4l-1
dd17352
otxA
NKX2-4
dd16472
islet1
nkx2.2
dd24743
Post-2c
dd12317
Post-2b
SIX1 (dd15471)
six3
dd7324
nk4
IRX-2 (dd11500)
nkx6-2
prep
POU2/3
six1/2-2
Neurons
Parenchyma
six1/2-1+
Pharynx
Intestine
Nephridia
Muscle
Epidermis
Expression
High
Low

Supplemental Figure 1, related to Figure 1. Analysis of sequenced S/G_2/M cells, sequenced G_0 cells, and *in silico* TF catalog construction.

(A) Cells from the X1 and X2 FACS gate, showing *smedwi-1*, *p4hb*, and *pcna* expression levels.

(B) Expression of neoblast (*smedwi-1*) and cell-cycle markers (*mcm5* and *pcna*) in filtered specialized neoblasts and post-mitotic progenitors.

(C) Fraction of neoblast classes organized by fragment from which cells originated (anterior-facing fragment or posterior-facing fragment).

(D) Percentage of epidermis fated and non-epidermis fated neoblasts (S/G_2/M cells) expressing individual or multiple genes. Epidermis fated defined as cells in "Epidermis" cluster of S/G_2/M cells.

(E and F) UMAP plot visualizing S/G_2/M cells expressing a single epidermal FSTF or coexpressing multiple epidermal FSTFs.

(G) Correlation values for tissue-specific FSTFs and tissue-specific differentiated markers in neural, intestinal, and protonephridia G_0 cells (*pax6A* and *ChAT* coexpression for neurons; *gata4/5/6-1* and dd_888 coexpression for intestine; *POU2/3* and dd_2920 coexpression for protonephridia).

(H) Construction of *in silico* TF catalog: All planarian transcripts from the transcriptome were translated *in silico* and searched via HHMER for Pfam DNA-binding domains (DBDs) to categorize transcripts into a TF catalog. Genes were further labeled as: "Known FSTF" (previously published as planarian FSTF); "Known TF" (previously published as planarian TF, but without characterized FSTF attribution); "TF" (Not published as planarian TF, but with blast hit to Human TF"); or "No Blast" (no planarian TF characterization and no human blast hit).

(I) Expression log two-fold enrichment for each TF among different neoblast classes.

(J) Heatmap showing expression specificity score for select homeodomain TFs among different neoblast classes.

Figure S2

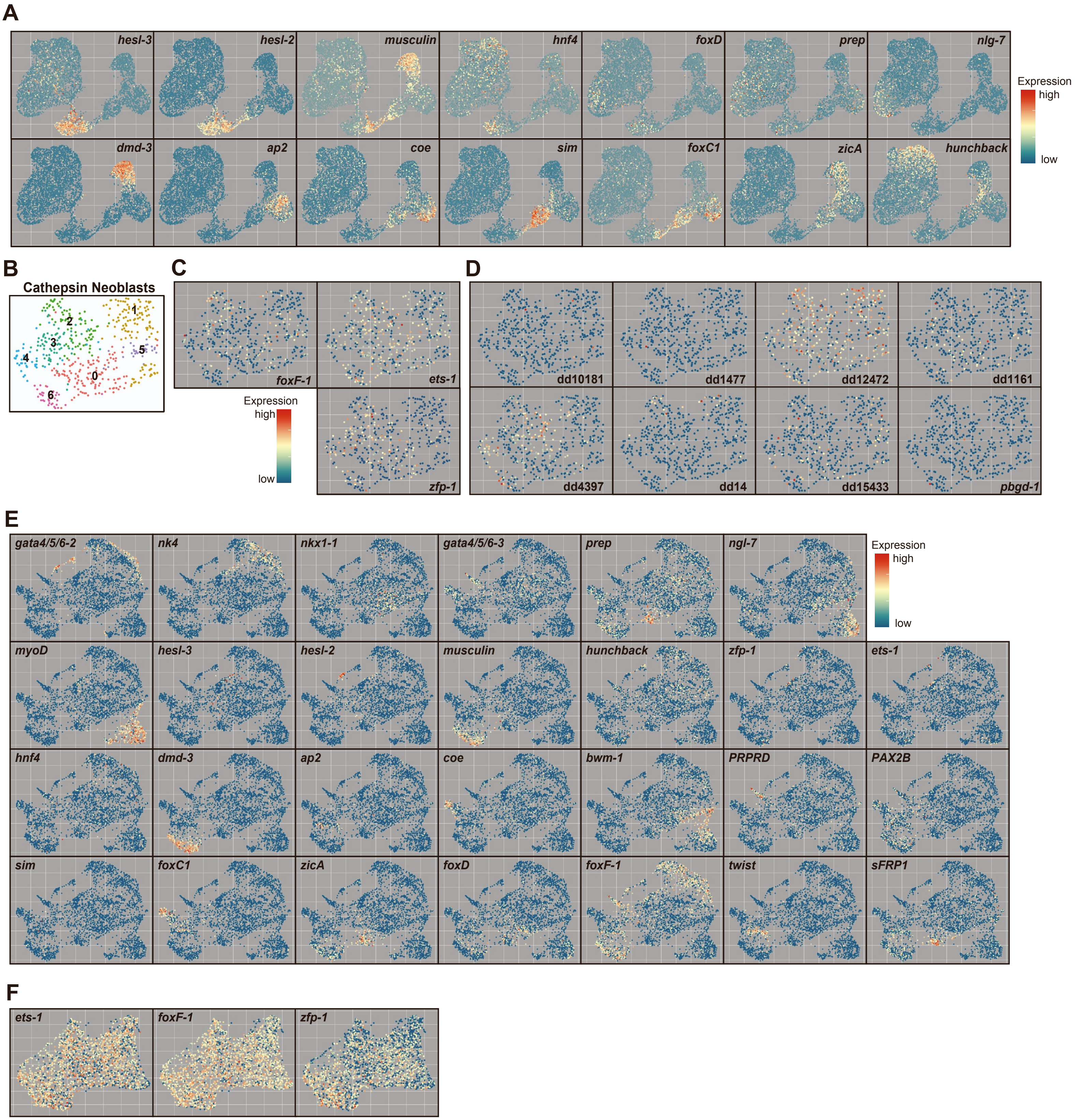
A
hesl-3
hesl-2
musculin
hnf4
foxD
prep
nlg-7
dmd-3
ap2
coe
sim
foxC1
zicA
hunchback
Expression
high
low
B
Cathepsin Neoblasts
0
1
2
3
4
5
6
C
foxF-1
ets-1
zfp-1
Expression
high
low
D
dd10181
dd1477
dd12472
dd1161
dd4397
dd14
dd15433
pbgd-1
E
gata4/5/6-2
nk4
nkx1-1
gata4/5/6-3
prep
ngl-7
Expression
high
low
myoD
hesl-3
hesl-2
musculin
hunchback
zfp-1
ets-1
hnf4
dmd-3
ap2
coe
bwm-1
PRPRD
PAX2B
sim
foxC1
zicA
foxD
foxF-1
twist
sFRP1
F
ets-1
foxF-1
zfp-1

Supplemental Figure 2, related to Figure 2. Expression of muscle and phagocytic transcription factors and genes in *PLOD1+* S/G_2/M cells, G_0 muscle cells, and G_0 *cathepsin*+ cells.

(A) UMAP plots visualizing expression of muscle-associated transcription factors (e.g., *hesl-3, musculin, foxD*), positional control genes (e.g., *nlg-7*), and phagocytic-associated transcription factors (e.g., *hunchback, hnf4*) in *PLOD1*+ S/G_2/M cells.

(B) "Cathepsin" cells from *PLOD1*+ S/G_2/M cells were subjected to an additional round of subclustering. UMAP plot of subclustered cathepsin-fated S/G_2/M cells.

(C) UMAP plots depict expression of phagocytic-associated transcription factors in subclustered cathepsin fated S/G_2/M cells.

(D) UMAP plots visualizing expression of subtype-specific phagocytic cell markers in *PLOD1+/cathepsin*+ S/G_2/M cells. Little neoblast diversity is apparent in cathepsin fated S/G_2/M cells.

(E) UMAP plots visualizing expression of muscle-associated transcription factors, positional control genes, and phagocytic-associated transcription factors in G_0 muscle cells.

(F) UMAP plots visualizing expression of phagocytic-associated transcription factors in cathepsin fated G_0 cells.

Figure S3

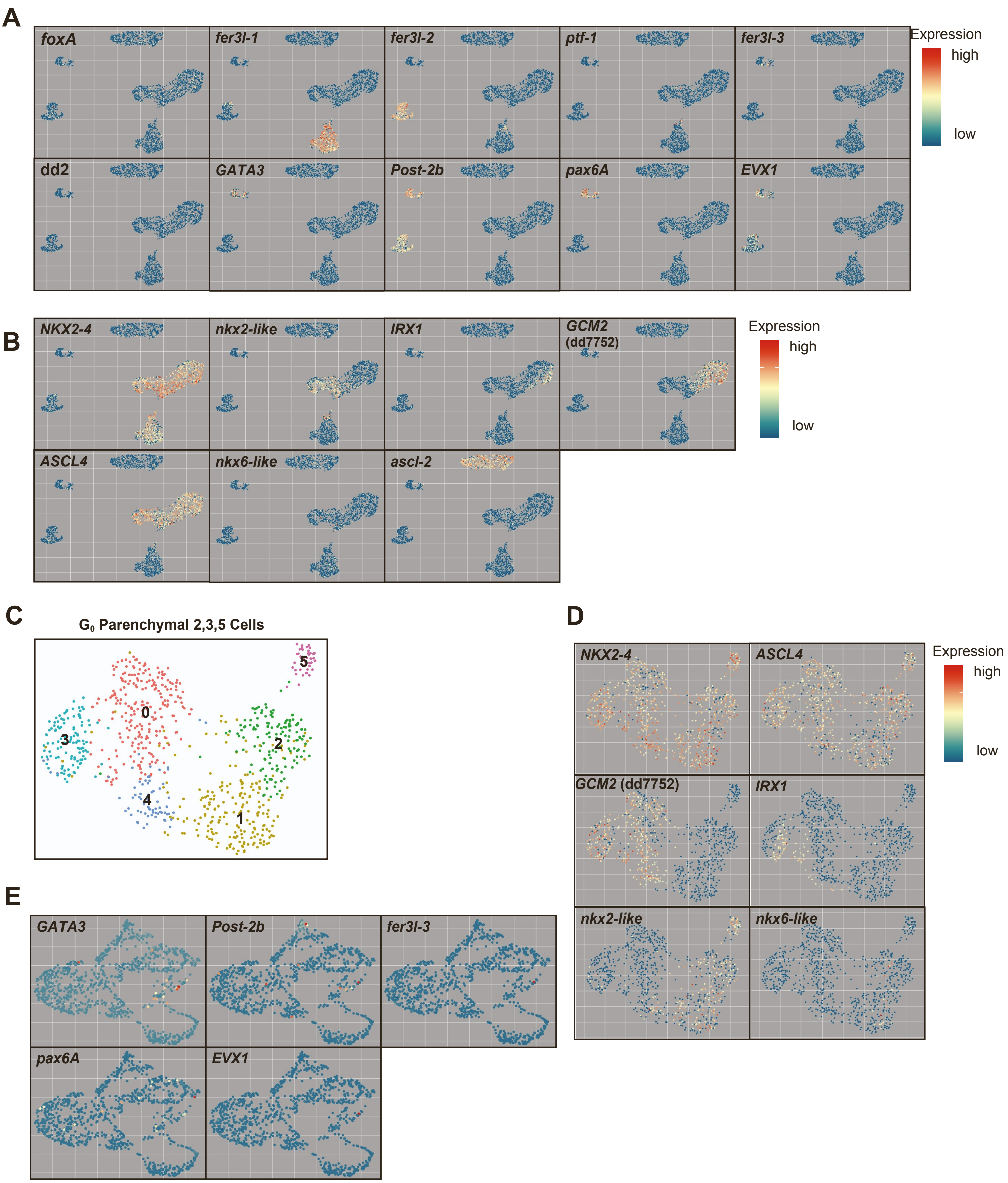
A
foxA
fer3l-1
fer3l-2
ptf-1
fer3l-3
dd2
GATA3
Post-2b
pax6A
EVX1
Expression
high
low
B
NKX2-4
nkx2-like
IRX1
GCM2
(dd7752)
ASCL4
nkx6-like
ascl-2
Expression
high
low
C
G0 Parenchymal 2,3,5 Cells
0
1
2
3
4
5
D
NKX2-4
ASCL4
GCM2 (dd7752)
IRX1
nkx2-like
nkx6-like
Expression
high
low
E
GATA3
Post-2b
fer3l-3
pax6A
EVX1

Supplemental Figure 3, related to Figure 3. Analysis of parenchymal S/G_2/M and G_0 cells.

(A and B) UMAP plot visualizing expression of TFs or other markers (e.g., dd_2) enriched in different subtypes of parenchymal G_0 cells.

(C) Subclusters 2, 3, and 5 of parenchymal G_0 cells were subjected to an additional round of subclustering together.

(D) UMAP plots depict expression of parenchymal-associated transcription factors in subclustered parenchymal G_0 cells from clusters 2, 3, and 5 specifically (combined).

(E) S/G_2/M parenchymal cell UMAP plot showing expression of transcription factors known to define one subtype of differentiated parenchymal cells (Fincher et al. 2018 scRNA-seq data clusters 6 and 10).

Figure S4

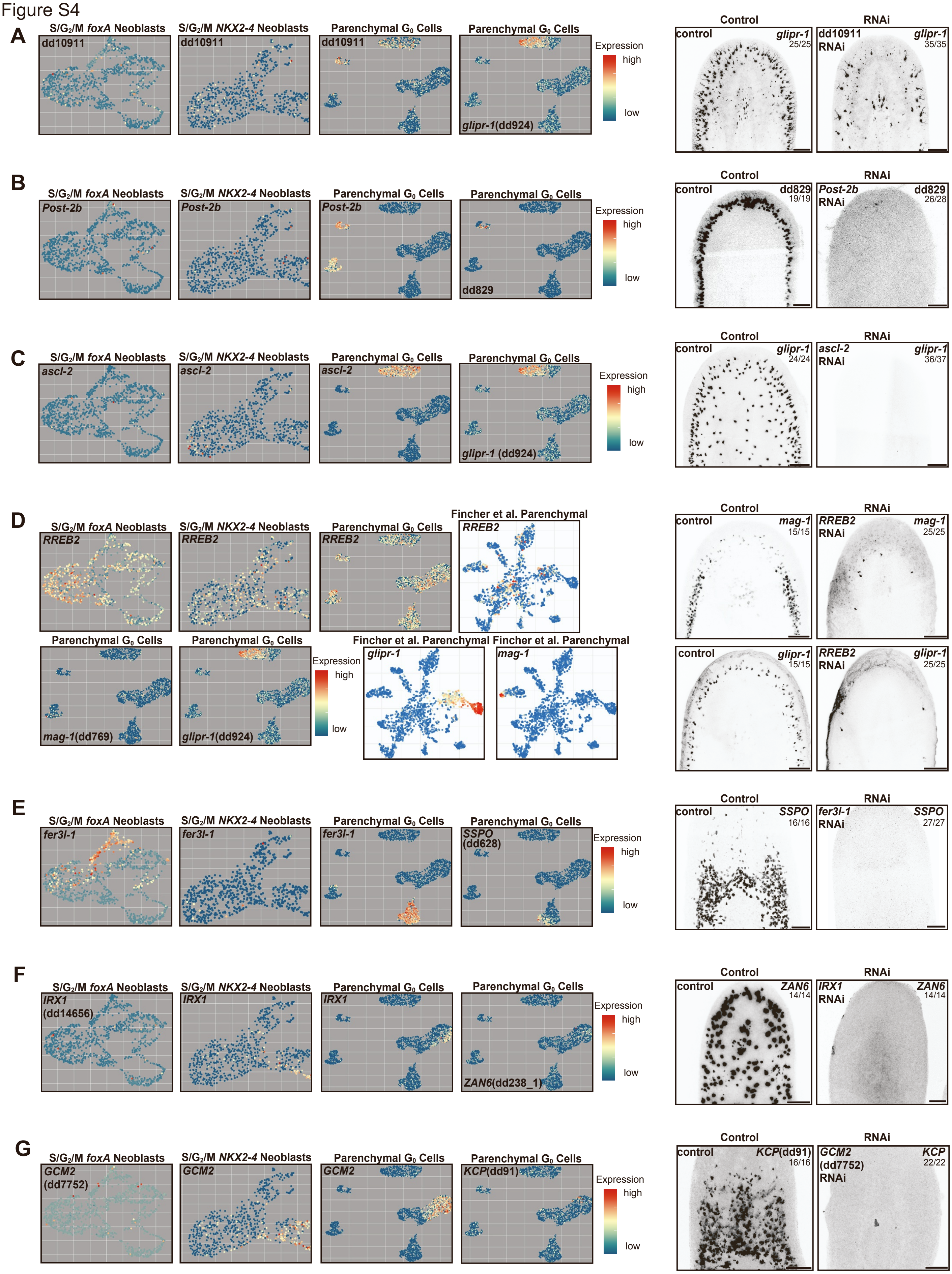
A
S/G2/M foxA Neoblasts
S/G2/M NKX2-4 Neoblasts
Parenchymal G0 Cells
Parenchymal G0 Cells
dd10911
dd10911
dd10911
glipr-1(dd924)
Expression
high
low
Control
RNAi
control
glipr-1
25/25
dd10911
RNAi
glipr-1
35/35
B
S/G2/M foxA Neoblasts
S/G2/M NKX2-4 Neoblasts
Parenchymal G0 Cells
Parenchymal G0 Cells
Post-2b
Post-2b
Post-2b
dd829
Expression
high
low
Control
RNAi
control
dd829
19/19
Post-2b
RNAi
dd829
26/28
C
S/G2/M foxA Neoblasts
S/G2/M NKX2-4 Neoblasts
Parenchymal G0 Cells
Parenchymal G0 Cells
ascl-2
ascl-2
ascl-2
glipr-1 (dd924)
Expression
high
low
Control
RNAi
control
glipr-1
24/24
ascl-2
RNAi
glipr-1
36/37
D
S/G2/M foxA Neoblasts
S/G2/M NKX2-4 Neoblasts
Parenchymal G0 Cells
Fincher et al. Parenchymal
RREB2
RREB2
RREB2
RREB2
Parenchymal G0 Cells
Parenchymal G0 Cells
mag-1(dd769)
glipr-1(dd924)
Expression
high
low
Fincher et al. Parenchymal
Fincher et al. Parenchymal
glipr-1
mag-1
Control
RNAi
control
mag-1
15/15
RREB2
RNAi
mag-1
25/25
control
glipr-1
15/15
RREB2
RNAi
glipr-1
25/25
E
S/G2/M foxA Neoblasts
S/G2/M NKX2-4 Neoblasts
Parenchymal G0 Cells
Parenchymal G0 Cells
fer3l-1
fer3l-1
fer3l-1
SSPO
(dd628)
Expression
high
low
Control
RNAi
control
SSPO
16/16
fer3l-1
RNAi
SSPO
27/27
F
S/G2/M foxA Neoblasts
S/G2/M NKX2-4 Neoblasts
Parenchymal G0 Cells
Parenchymal G0 Cells
IRX1
(dd14656)
IRX1
IRX1
ZAN6(dd238_1)
Expression
high
low
Control
RNAi
control
ZAN6
14/14
IRX1
RNAi
ZAN6
14/14
G
S/G2/M foxA Neoblasts
S/G2/M NKX2-4 Neoblasts
Parenchymal G0 Cells
Parenchymal G0 Cells
GCM2
(dd7752)
GCM2
GCM2
KCP(dd91)
Expression
high
low
Control
RNAi
control
KCP(dd91)
16/16
GCM2
(dd7752)
RNAi
KCP
22/22

Supplemental Figure 4, related to Figure 3. Functional characterization of parenchymal enriched TFs in cell fate specification.

(A) Left: expression of dd_10911 (TF-encoding gene) in all parenchymal S/G_2/M cells (*foxA*+ population and *NKX2-4* population) and all parenchymal G_0 cells. Expression of *glipr1* (dd_924) in parenchymal G_0 cells. Right: RNAi showing loss of *glipr1*+ cells following dd_10911 RNAi.

(B) Left: expression of *Post-2b* in all parenchymal S/G_2/M cells (*foxA*+ population and *NKX2-4* population) and all parenchymal G_0 cells. Expression of (dd_829) in parenchymal G_0 cells. Right: RNAi showing loss of dd_829 cells following *Post-2b* RNAi.

(C) Left: expression of *ascl-2* in all parenchymal S/G_2/M cells (*foxA*+ population and *NKX2-4* population) and all parenchymal G_0 cells. Expression of *glipr1* in parenchymal G_0 cells. Right: RNAi showing loss of *glipr1*+ cells following *ascl-2* RNAi.

(D) Left: expression of genes encoding parenchymal-associated TFs (*RREB2*) and differentiated parenchymal subtype markers (*mag-1*, *glipr-1*) in all parenchymal S/G_2/M cells (*foxA*+ population and *NKX2-4* population), all parenchymal G_0 cells, and all parenchymal cells from Fincher et al. 2018 data. Right: RNAi showing loss of *glipr1*+ cells and *mag-1*+ cells following *RREB2* RNAi.

(E) Left: expression of *ferl3-1* in all parenchymal S/G_2/M cells (*foxA*+ population and *NKX2-4* population) and all parenchymal G_0 cells. Expression of *SSPO* (dd_628) in parenchymal G_0 cells. Right: RNAi showing loss of *SSPO*+ cells following *fer3l-1* RNAi.

(F) Left: expression of *IRX1* (dd_14656) in all parenchymal S/G_2/M cells (*foxA*+ population and *NKX2-4* population) and all parenchymal G_0 cells. Expression of *ZAN6* (dd_238_1) in parenchymal G_0 cells. Right: RNAi showing loss of *ZAN6*+ cells following *IRX1* RNAi.

(G) Left: expression of *GCM2* (dd_7752) in all parenchymal S/G_2/M cells (*foxA*+ population and *NKX2-4* population) and all parenchymal G_0 cells. Expression of *KCP* (dd_91) in parenchymal G_0 cells. Right: RNAi showing loss of *KCP*+ cells following *GCM2* RNAi.

(A-G) FISH images are the same as in Figure 3J, but zoomed out. Number of animals with scored phenotype indicated by fraction below marker gene name. Scale bars are 100 micrometers.

Figure S5

A

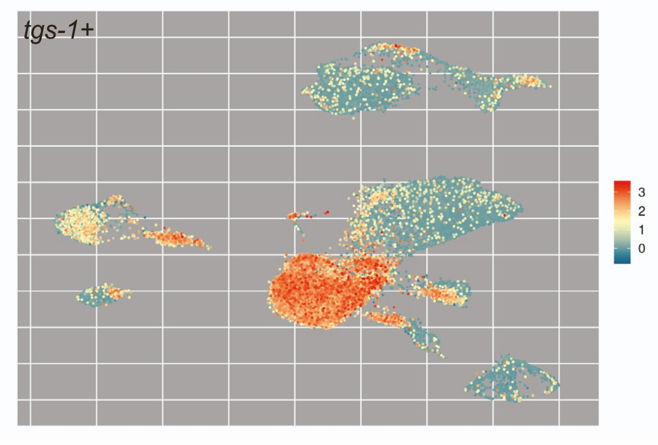

B

FSTF	FSTF id	% inside Cluster 0	% outside Cluster 0
ski-3	dd10394	20.38002	2.075287
nkx6	dd11198	6.328532	0.8819969
tcf-1	dd13056	9.806926	1.147172
nkx2-like	dd13898	5.914802	1.152937
pitx	dd15253	2.850138	1.210584
otxb	dd15516	1.777505	0.3977633
pou4l-1	dd15555	10.26663	1.227878
runt1	dd16222	22.75513	6.934917
sp6-9	dd17385	13.48452	1.10682
pax6A	dd17726	13.08612	1.533406
scratch	dd18952	10.14404	1.838935
otp	dd19326	2.727551	0.7782325
neuroD-1	dd21717	7.585044	0.8301147
pax6B	dd35892	2.114618	0.5476451
ovo	dd48430	0.6129329	0.3401164
sox2	dd8104	3.279191	1.792817
coe	dd9892	2.773521	2.305874
Zinc F.	dd10911	20.53325	5.718568

Percent of *cluster 0* cells with 1+ Neural FSTF: **78%**

Percent of *non-cluster 0* cells with 1+ Neural FSTF: **25%**

Percent of *tgs+* cells with 1+ Neural FSTF: **65%**

Percent of *tgs-* cells with 1+ Neural FSTF: **24%**

C

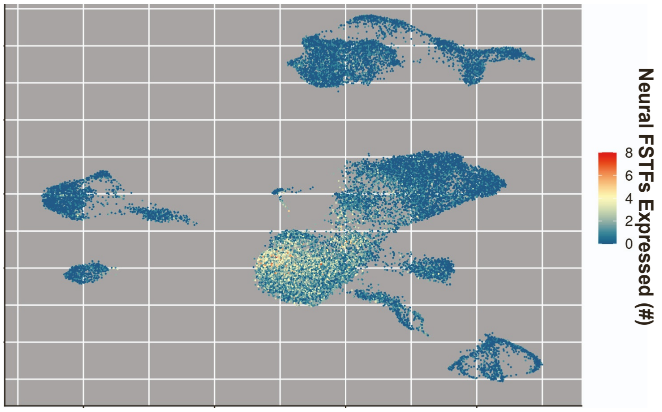

D

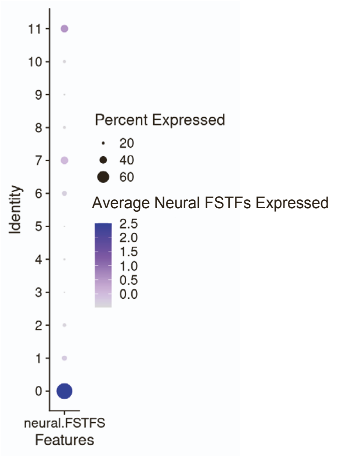

E

FSTF	FSTF id	% inside PLOD clusters	% outside PLOD clusters
myoD	dd12634	10.0	0.6
lbx	dd31425	2.7	0.2
nk4	dd19327	5.2	1.0
nkx1-1	dd19327	2.1	0.2
gata4/5/6-2	dd9910	9.6	0.8
gata4/5/6-3	dd8208	3.3	1.5
musculin	dd16070	16.3	0.7
twist	dd17951	8.5	0.3
ets-1	dd2092	8.3	0.6
foxF-1	dd6910	14.8	1.0
hesl-2	dd17332	8.3	0.5

Percent of *PLOD cluster* cells with 1+ Neural FSTF: **61%**

Percent of *non-PLOD cluster* cells with 1+ Neural FSTF: **7.2%**

Supplemental Figure 5, related to Figure 4. Analysis of *tgs-1*+ neoblast population and neural specification

(A) *tgs-1*+ expression in S/G_2/M neoblasts.

(B) Percentage of cells expressing select neural FSTFs in *tgs-1*-enriched cluster 0 and outside of that cluster. Summary statistics of cells expressing at least one neural FSTF in either *tgs-1*+ cells, regardless of cluster, or in cluster 0.

(C) UMAP plot of cells by the number of select neural FSTFs expressed in each cell.

(D) Dot plot of clusters by the number of select neural FSTFs expressed in cells within that cluster.

(E) Percentage of cells expressing select muscle and cathepsin cell FSTFs in *PLOD1*-enriched clusters 2, 4, and 9 and outside of those clusters. Summary statistics of cells expressing at least one muscle/cathepsin cell FSTF. For comparison to neural/cluster 0.

Figure S6

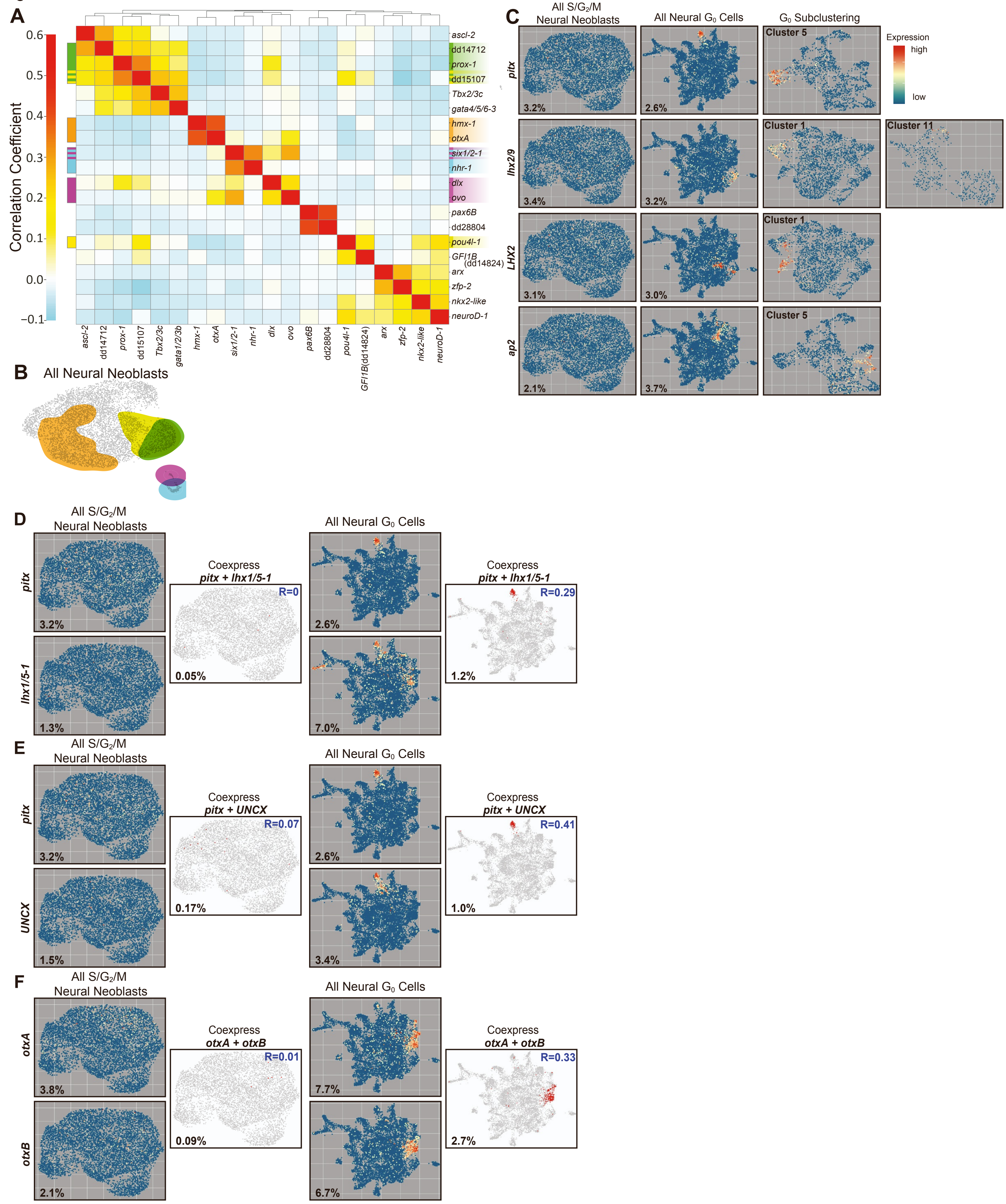
A
Correlation Coefficient
0.6
0.5
0.4
0.3
0.2
0.1
0.0
−0.1
ascl-2
dd14712
prox-1
dd15107
Tbx2/3c
gata4/5/6-3
hmx-1
otxA
six1/2-1
nhr-1
dlx
ovo
pax6B
dd28804
pou4l-1
GFI1B (dd14824)
arx
zfp-2
nkx2-like
neuroD-1
gata1/2/3b
GFI1B(dd14824)
B
All Neural Neoblasts
C
All S/G2/M Neural Neoblasts
All Neural G0 Cells
G0 Subclustering
Expression
high
low
pitx
lhx2/9
LHX2
ap2
Cluster 5
Cluster 1
Cluster 11
3.2%
2.6%
3.4%
3.2%
3.1%
3.0%
2.1%
3.7%
D
All S/G2/M Neural Neoblasts
All Neural G0 Cells
Coexpress pitx + lhx1/5-1
lhx1/5-1
R=0
R=0.29
0.05%
1.2%
1.3%
7.0%
E
Coexpress pitx + UNCX
UNCX
R=0.07
R=0.41
0.17%
1.0%
1.5%
3.4%
F
Coexpress otxA + otxB
otxA
otxB
R=0.01
R=0.33
3.8%
0.09%
7.7%
2.7%
2.1%
6.7%

Supplemental Figure 6, related to Figure 4. Neural FSTF expression in neural progenitors (S/G_2**/M and** G_0**).**

(A) Correlation analysis between transcription factors showing TF modules for all S/G_2/M neural subtypes ("Neural" and "*six1/2-1+"* cells combined). Modules were determined by computing pairwise correlations between all transcription factors (from TF catalog) of all S/G_2/M neural cells ("Neural" and "*six1/2-1+"* cells).

(B) Visualization of TF module expression domain on UMAP plot for all S/G_2/M cells from "Neural" and "*six1/2-1+*" clusters.

(C) Expression of neural FSTFs in neural S/G_2/M cells, G_0 cells, and respective G_0 subclusters. Measurements in lower left of boxes refer to percentages of cells expressing a given FSTF among noted neural progenitors.

(D, E, and F) Expression of individual neural TFs and coexpression of multiple neural TFs in neural S/G_2/M cells and neural G_0 cells. FSTFs defining unique subtypes of neurons are expressed but lowly-to-not correlated in S/G_2/M cells, and become substantially correlated in the G_0 state.

Figure S7

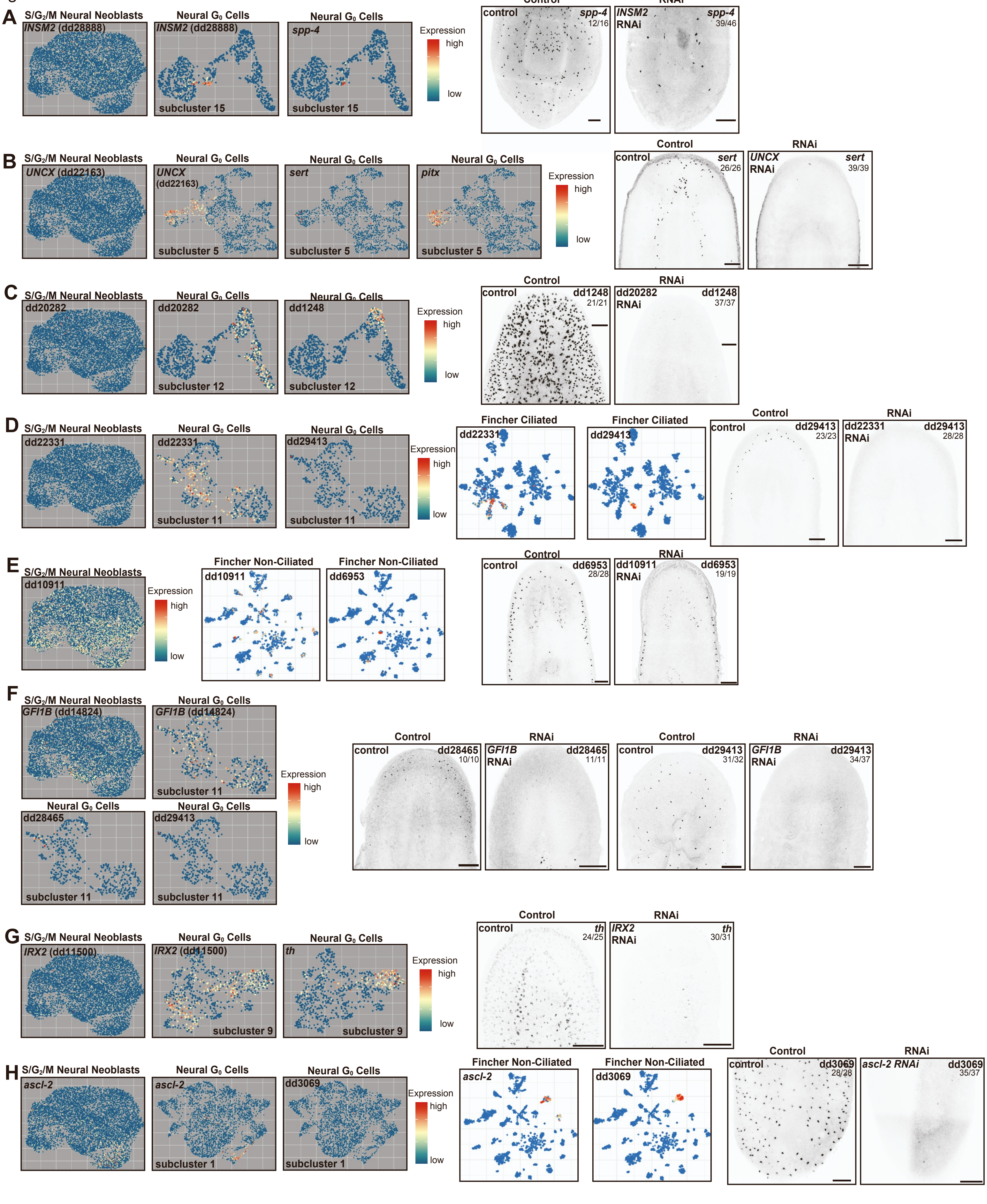
A
S/G_2/M Neural Neoblasts
INSM2 (dd28888)
Neural G_0 Cells
INSM2 (dd28888)
subcluster 15
Neural G_0 Cells
spp-4
subcluster 15
Expression
high
low
Control
control
spp-4
12/16
RNAi
INSM2
RNAi
spp-4
39/46
B
S/G_2/M Neural Neoblasts
UNCX (dd22163)
Neural G_0 Cells
UNCX
(dd22163)
subcluster 5
Neural G_0 Cells
sert
subcluster 5
Neural G_0 Cells
pitx
subcluster 5
Expression
high
low
Control
control
sert
26/26
RNAi
UNCX
RNAi
sert
39/39
C
S/G_2/M Neural Neoblasts
dd20282
Neural G_0 Cells
dd20282
subcluster 12
Neural G_0 Cells
dd1248
subcluster 12
Expression
high
low
Control
control
dd1248
21/21
RNAi
dd20282
RNAi
dd1248
37/37
D
S/G_2/M Neural Neoblasts
dd22331
Neural G_0 Cells
dd22331
subcluster 11
Neural G_0 Cells
dd29413
subcluster 11
Expression
high
low
Fincher Ciliated
dd22331
Fincher Ciliated
dd29413
Control
control
dd29413
23/23
RNAi
dd22331
RNAi
dd29413
28/28
E
S/G_2/M Neural Neoblasts
dd10911
Expression
high
low
Fincher Non-Ciliated
dd10911
Fincher Non-Ciliated
dd6953
Control
control
dd6953
28/28
RNAi
dd10911
RNAi
dd6953
19/19
F
S/G_2/M Neural Neoblasts
GFI1B (dd14824)
Neural G_0 Cells
GFI1B (dd14824)
subcluster 11
Neural G_0 Cells
dd28465
subcluster 11
Neural G_0 Cells
dd29413
subcluster 11
Expression
high
low
Control
control
dd28465
10/10
RNAi
GFI1B
RNAi
dd28465
11/11
Control
control
dd29413
31/32
RNAi
GFI1B
RNAi
dd29413
34/37
G
S/G_2/M Neural Neoblasts
IRX2 (dd11500)
Neural G_0 Cells
IRX2 (dd11500)
subcluster 9
Neural G_0 Cells
th
subcluster 9
Expression
high
low
Control
control
th
24/25
RNAi
IRX2
RNAi
th
30/31
H
S/G_2/M Neural Neoblasts
ascl-2
Neural G_0 Cells
ascl-2
subcluster 1
Neural G_0 Cells
dd3069
subcluster 1
Expression
high
low
Fincher Non-Ciliated
ascl-2
Fincher Non-Ciliated
dd3069
Control
control
dd3069
28/28
RNAi
ascl-2 RNAi
dd3069
35/37

Supplemental Figure 7, related to Figure 4. Role of novel neural FSTFs in fate specification of distinct neural subtypes.

(A) Left: expression of *INSM2* in neural S/G_2/M cells and a subset of neural G_0 cells expressing *spp-4*. Right: RNAi showing loss of *spp-4*+ neurons following *INSM2* RNAi.

(B) Left: expression of *UNCX* in neural S/G_2/M cells and in serotonergic G_0 cells (defined by *sert* and *pitx* coexpression). Right: RNAi showing loss of serotonergic neurons (*sert*+) following *UNCX* RNAi.

(C) Left: expression of dd_20282 (TF-encoding gene) in neural S/G_2/M cells and in a subset of non-ciliated peripheral neural G_0 cells defined by dd_1248 expression. Right: RNAi showing loss of dd_1248+ non-ciliated peripheral neurons following dd_20282 RNAi.

(D) Left: expression of dd_22331 (TF-encoding gene) in neural S/G_2/M cells and subset of ciliated peripheral neural G_0 cells defined by dd_29413 expression. Right: Expression of dd_22331 in a subset of ciliated neurons from Fincher et al. 2018 data. RNAi showing loss of dd_29413+ ciliated peripheral neurons following dd_22331 RNAi.

(E) Left: expression of dd_10911 (TF-encoding gene) in neural S/G_2/M cells. Expression of dd_10911 and dd_6953 (differentiated neural subtype marker) in non-ciliated cells of Fincher et al. 2018 scRNA-seq data. Right: RNAi showing loss of dd_6953+ cells following dd_10911 RNAi.

(F) Left: expression of *GFI1B* in neural S/G_2/M cells and a subset of ciliated peripheral neural G_0 cells defined by dd_29413 expression and dd_28465 expression. Right: RNAi showing loss of dd_29413+ ciliated peripheral neurons and dd_28465 neurons following *GFI1B* RNAi.

(G) Left: expression of *IRX2* in neural S/G_2/M cells and in subset of dopaminergic G_0 cells (defined by *tyrosine hydroxylase (th)* expression). Right: RNAi showing loss of dopaminergic neurons (*th*+) following *IRX2* RNAi.

(H) Left: expression of *aslc-2* in neural S/G_2/M cells and in a subset of non-ciliated peripheral G_0 neurons defined by dd_3069 expression. Right: Expression of *ascl*-2 in a subset of non-niliated

neurons from Fincher et al. 2018 data. RNAi showing loss of dd_3069+ non-ciliated peripheral neurons following *ascl-2* RNAi.

(A-H) FISH images are the same as in Figure 4E, but zoomed out. Number of animals with scored phenotype indicated by fraction below marker gene name. Scale bars are 100 micrometers.

Figure S8

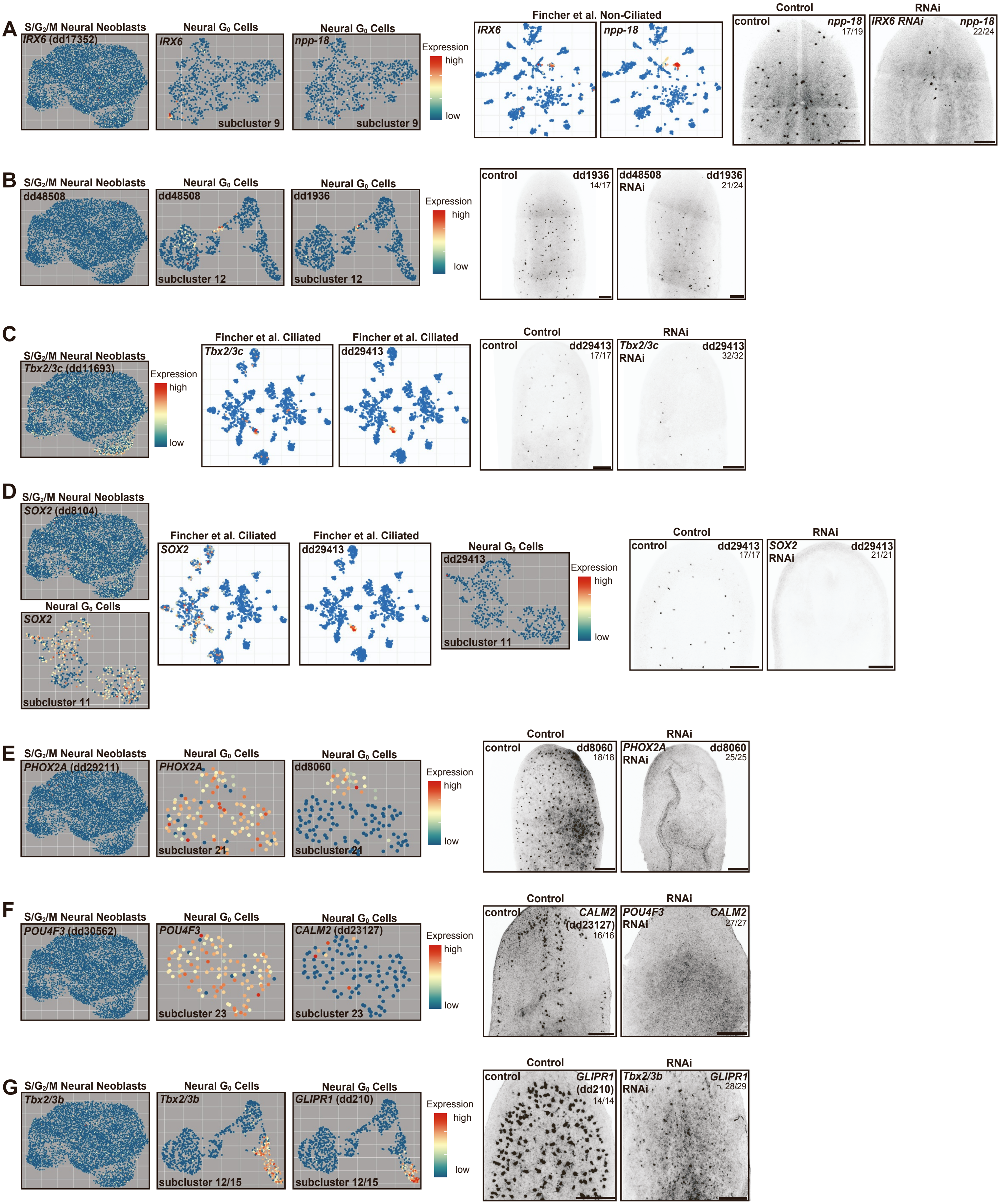

A
S/G2/M Neural Neoblasts
IRX6 (dd17352)
Neural G0 Cells
IRX6
subcluster 9
Neural G0 Cells
npp-18
subcluster 9
Expression
high
low
Fincher et al. Non-Ciliated
IRX6
npp-18
Control
control
npp-18
17/19
RNAi
IRX6 RNAi
npp-18
22/24
B
S/G2/M Neural Neoblasts
dd48508
Neural G0 Cells
dd48508
subcluster 12
Neural G0 Cells
dd1936
subcluster 12
Expression
high
low
control
dd1936
14/17
dd48508
RNAi
dd1936
21/24
C
S/G2/M Neural Neoblasts
Tbx2/3c (dd11693)
Expression
high
low
Fincher et al. Ciliated
Tbx2/3c
Fincher et al. Ciliated
dd29413
Control
control
dd29413
17/17
RNAi
Tbx2/3c
RNAi
dd29413
32/32
D
S/G2/M Neural Neoblasts
SOX2 (dd8104)
Neural G0 Cells
SOX2
subcluster 11
Fincher et al. Ciliated
SOX2
Fincher et al. Ciliated
dd29413
Neural G0 Cells
dd29413
subcluster 11
Expression
high
low
Control
control
dd29413
17/17
RNAi
SOX2
RNAi
dd29413
21/21
E
S/G2/M Neural Neoblasts
PHOX2A (dd29211)
Neural G0 Cells
PHOX2A
subcluster 21
Neural G0 Cells
dd8060
subcluster 21
Expression
high
low
Control
control
dd8060
18/18
RNAi
PHOX2A
RNAi
dd8060
25/25
F
S/G2/M Neural Neoblasts
POU4F3 (dd30562)
Neural G0 Cells
POU4F3
subcluster 23
Neural G0 Cells
CALM2 (dd23127)
subcluster 23
Expression
high
low
Control
control
CALM2
(dd23127)
16/16
RNAi
POU4F3
RNAi
CALM2
27/27
G
S/G2/M Neural Neoblasts
Tbx2/3b
Neural G0 Cells
Tbx2/3b
subcluster 12/15
Neural G0 Cells
GLIPR1 (dd210)
subcluster 12/15
Expression
high
low
Control
control
GLIPR1
(dd210)
14/14
RNAi
Tbx2/3b
RNAi
GLIPR1
28/29

Supplemental Figure 8, related to Figure 4. Role of novel neural FSTFs in fate specification of distinct neural subtypes.

(A) Left: expression of *IRX6* in neural S/G_2/M cells and in *npp-18*+ G_0 neurons. Right: Expression of *IRX6* in non-ciliated neurons from Fincher et al. 2018 data. RNAi showing loss of *npp-18*+ neurons following *IRX6* RNAi.

(B) Left: expression of dd_48508 (TF-encoding gene) in a subset of non-ciliated neurons defined by dd_1936 expression. Right: RNAi showing loss of dd_1936+ non-ciliated neurons following dd_48508 RNAi.

(C) Left: expression of *Tbx2/3c* in neural S/G_2/M cells. Expression of *Tbx2/3c* and dd_29413 in a subcluster of ciliated neurons from Fincher et al. 2018 scRNA-seq data. Right: RNAi showing loss of dd_29413+ ciliated neurons following *Tbx2/3c* RNAi.

(D) Left: expression of *SOX2* in neural S/G_2/M cells and a subset of dd_29413+ neural G_0 cells. Expression of SOX2 in a subset of ciliated neurons from Fincher et al. 2018 data. Right: RNAi showing loss of dd_29413+ ciliated neurons following *SOX2* RNAi.

(E) Left: expression of *PHOX2A* in neural S/G_2/M cells and a subset of dd_8060+ neural G_0 cells. Right: RNAi showing loss of dd_8060+ neurons following *PHOX2A* RNAi.

(F) Left: expression of *POU4F3* in neural S/G_2/M cells and a subset of *CALM2*+ neural G_0 cells. Right: RNAi showing loss of *CALM2*+ neurons following *POU4F3* RNAi.

(G) Left: expression of *Tbx2/3b* in neural S/G_2/M cells and a subset of *GLIPR1*+ neural G_0 cells. Right: RNAi showing loss of *GLIPR1*+ neurons following *Tbx2/3b* RNAi.

(A-G) FISH images are the same as in Figure 4E, but zoomed out. Number of animals with scored phenotype indicated by fraction below marker gene name. Scale bars are 100 micrometers.

Figure S9

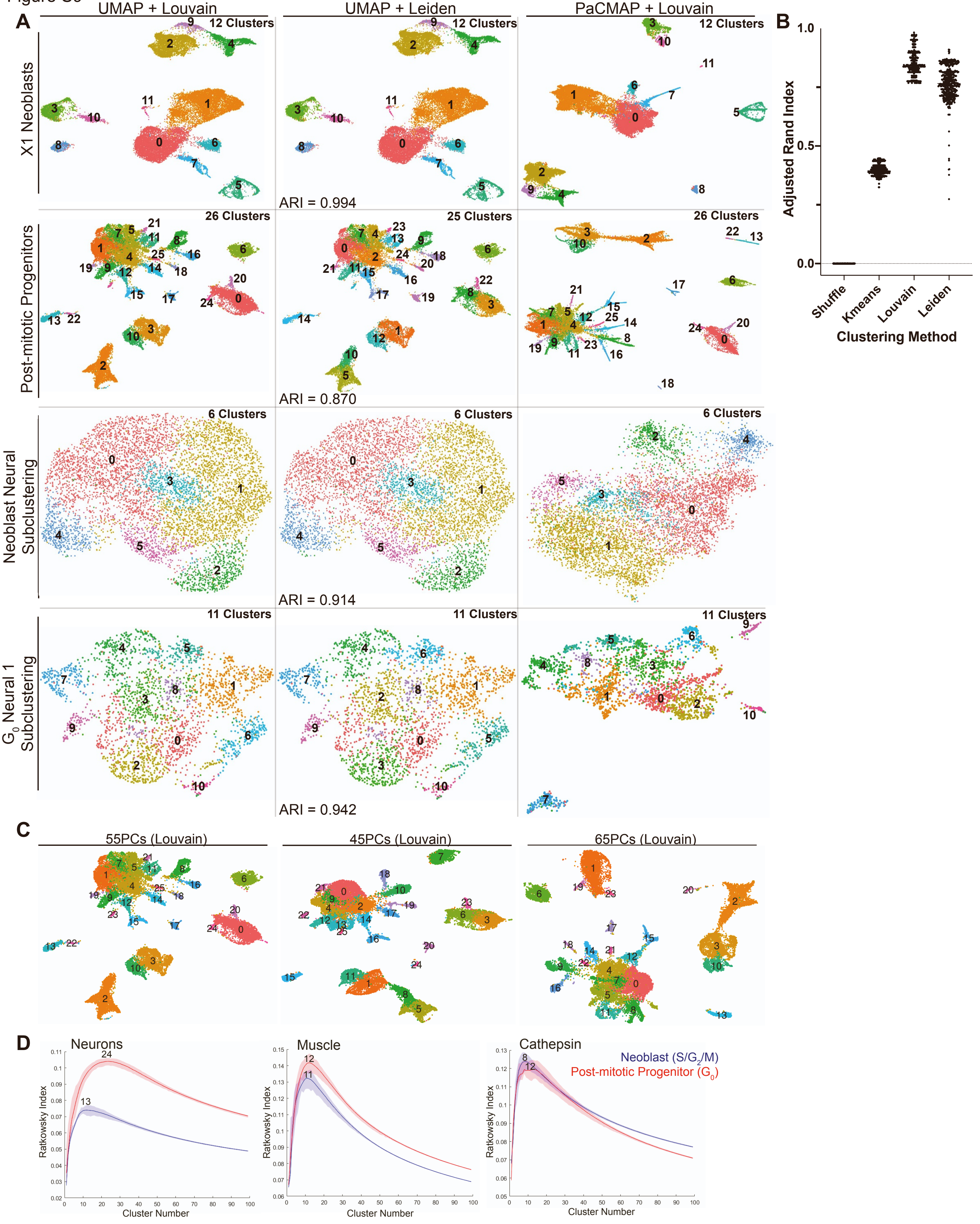
A
UMAP + Louvain
UMAP + Leiden
PaCMAP + Louvain
X1 Neoblasts
12 Clusters
ARI = 0.994
Post-mitotic Progenitors
26 Clusters
25 Clusters
ARI = 0.870
Neoblast Neural Subclustering
6 Clusters
ARI = 0.914
G_0 Neural 1 Subclustering
11 Clusters
ARI = 0.942
B
Adjusted Rand Index
Shuffle
Kmeans
Louvain
Leiden
Clustering Method
C
55PCs (Louvain)
45PCs (Louvain)
65PCs (Louvain)
D
Neurons
Muscle
Cathepsin
Ratkowsky Index
Cluster Number
Neoblast (S/G_2/M)
Post-mitotic Progenitor (G_0)

Supplemental Figure 9, related to Figure 4 and 1. Robustness of clustering, visualization, and analysis methods.

(A) Comparison between UMAP and PaCMAP for cell visualization and Louvain and Leiden-based clustering methods for neoblasts, post-mitotic progenitors, neural neoblast subclustering, and neural post-mitotic progenitor cluster 1 subclustering. Adjusted Rand Index is given in bottom left for cluster membership comparison. Number of clusters generated is given in top right.

(B) Adjusted Rand Index distributions for data clustered through different generations of random shuffling, k-means clustering, Louvain clustering, and Leiden clustering compared to the initial Louvain clustering of post-mitotic progenitors.

(C) Comparison between Louvain clusterings of post-mitotic progenitors using different numbers of top principal components in the analysis.

(D) Ratkowsky index (the number of groups that best explain differences between cells) for different tissue types during the neoblast ($S/G_2/M$) and post-mitotic (G_0) stages. The number of groups that best explain differences between cells is roughly matched in neoblast and post-mitotic states for muscle and *cathepsin*+ tissues, but greatly expanded for neurons. Numbers above each curve note the number of groups with the highest Ratkowsky index.

Figure S10

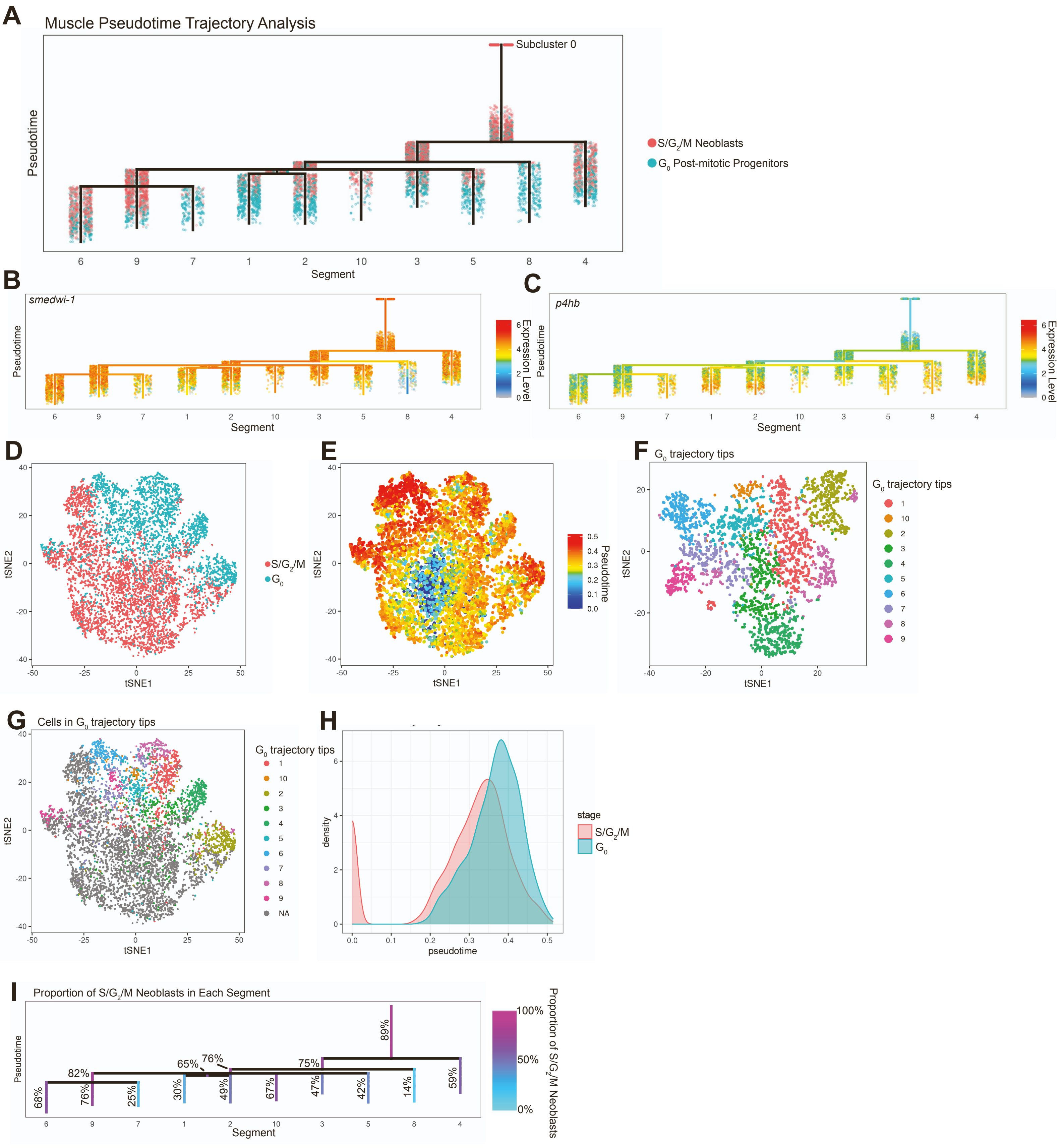

A
Muscle Pseudotime Trajectory Analysis
Subcluster 0
Pseudotime
Segment
S/G2/M Neoblasts
G0 Post-mitotic Progenitors
B
smedwi-1
C
p4hb
Expression Level
D
tSNE1
tSNE2
S/G2/M
G0
E
Pseudotime
F
G0 trajectory tips
G
Cells in G0 trajectory tips
H
density
pseudotime
stage
I
Proportion of S/G2/M Neoblasts in Each Segment
68%
82%
76%
25%
30%
65%
76%
49%
67%
75%
47%
42%
14%
89%
59%
100%
50%
0%
Proportion of S/G2/M Neoblasts

Supplemental Figure 10, related to Figure 2. URD transcriptional pseudotime trajectory analysis for muscle

(A) Transcriptional pseudotime trajectory for muscle-specified neoblasts and G_0 cells, rooted by muscle neoblast subcluster 0.

(B) Expression of *smedwi-1* and (C) *p4hb* along pseudotime trajectories show gradients along pseudotime.

(D and E) tSNE dimensionality reduction of all muscle-specified cells included and their corresponding pseudotime (E).

(F and G) Louvain clustering of G_0 cells used as trajectory tips and their corresponding positions in tSNE space with specified neoblasts (G).

(H) Pseudotime distributions for $S/G_2/M$ neoblasts and G_0 post-mitotic progenitors.

(I) Percentages of $S/G_2/M$ neoblasts in each trajectory segment shows neoblasts present in all segments, including terminal segments with G_0 cells.

Figure S11

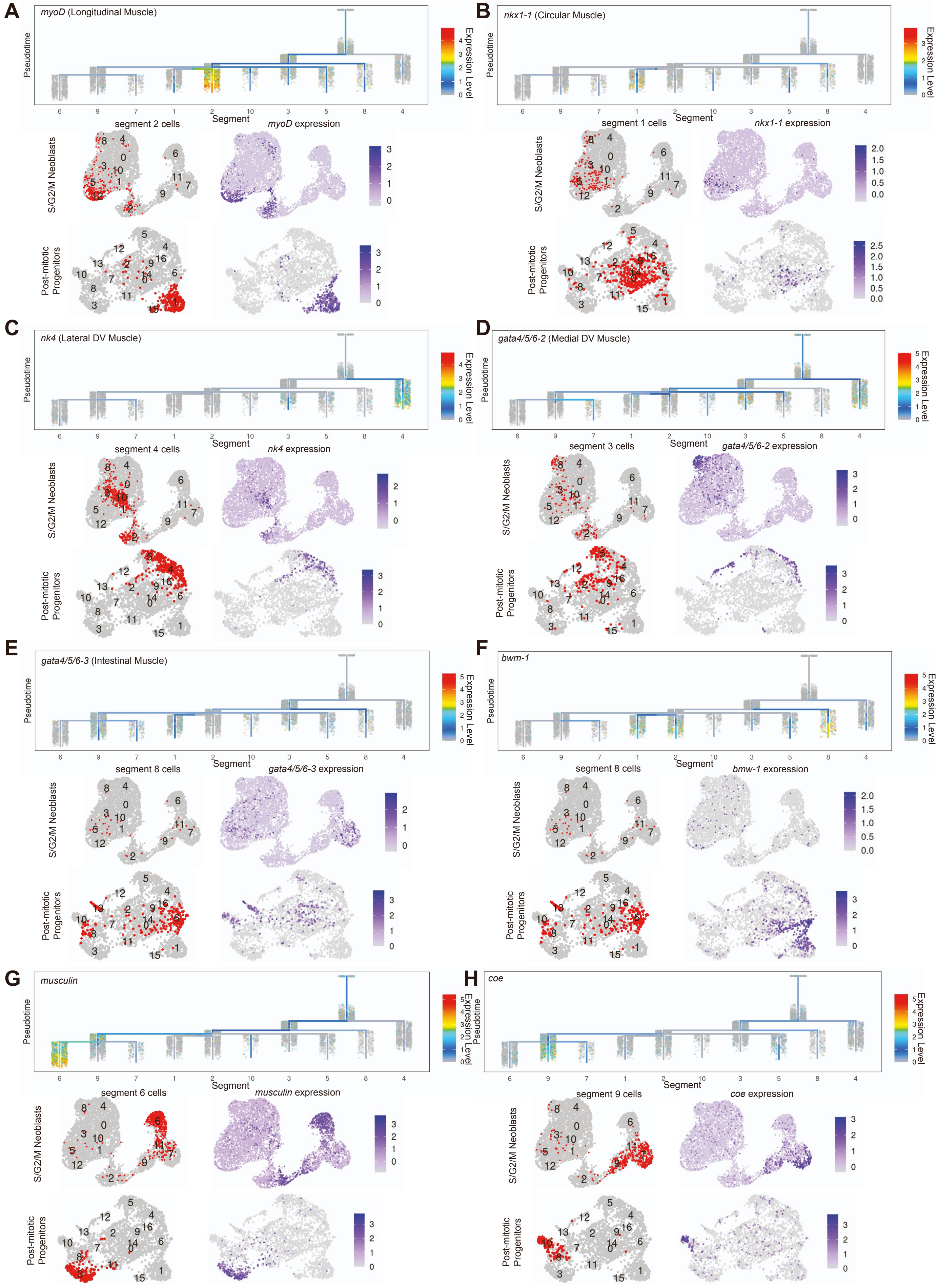

A
myoD (Longitudinal Muscle)
Pseudotime
Expression Level
Segment
segment 2 cells
myoD expression
S/G2/M Neoblasts
Post-mitotic Progenitors
B
nkx1-1 (Circular Muscle)
segment 1 cells
nkx1-1 expression
C
nk4 (Lateral DV Muscle)
segment 4 cells
nk4 expression
D
gata4/5/6-2 (Medial DV Muscle)
segment 3 cells
gata4/5/6-2 expression
E
gata4/5/6-3 (Intestinal Muscle)
segment 8 cells
gata4/5/6-3 expression
F
bwm-1
segment 8 cells
bmw-1 expression
G
musculin
segment 6 cells
musculin expression
H
coe
segment 9 cells
coe expression

Supplemental Figure 11, related to Figure 2. Muscle cell-type pseudotime trajectories from URD analysis

(A-Q) Pseudotime trajectories of identified muscle subtypes.

(A) Expression of *myoD* is enriched in segment 2 (top), with cells in segment 2 existing in previously identified longitudinal muscle-specified clusters (bottom-left), enriched in *myoD* (bottom-right).

(B) Expression of *nkx1-1* is enriched in segment 1 (top), with cells in segment 1 existing in previously identified circular fiber-specified clusters (bottom-left), enriched in *nkx1-1* (bottom-right).

(C) Expression of *nk4* is enriched in segment 4 (top), with cells in segment 4 existing in previously identified circular fiber-specified clusters (bottom-left), enriched in *nk4* (bottom-right).

(D) Expression of *gata4/5/6-2* is enriched in segment 3 (top), with cells in segment 3 existing in previously identified medial DV muscle-specified clusters (bottom-left), enriched in *gata4/5/6-2* (bottom-right).

(E) Expression of *gata4/5/6-3* is enriched in segment 8 (top), with cells in segment 8 existing in previously identified intestinal muscle-specified clusters (bottom-left), enriched in *gata4/5/6-3* (bottom-right).

(F) Segment 8 also is enriched in the expression of *bwm-1* (top), with cells in segment 8 also existing in a previously identified G_0 clustered enriched in *bwm*-1 (bottom).

Pseudotime trajectories of identified pharynx muscle subtypes.

(G) Pseudotime trajectories of identified pharynx muscle subtypes. Expression of *musculin* is enriched in segment 6 (top), with cells in segment 6 existing in previously identified pharynx muscle-specified clusters (bottom-left), enriched in *musculin* (bottom-right).

(H) Expression of *coe* is enriched in segment 9 (top), with cells in segment 9 existing in previously identified pharynx muscle-specified clusters (bottom-left), enriched in *coe* (bottom-right).

Figure S12

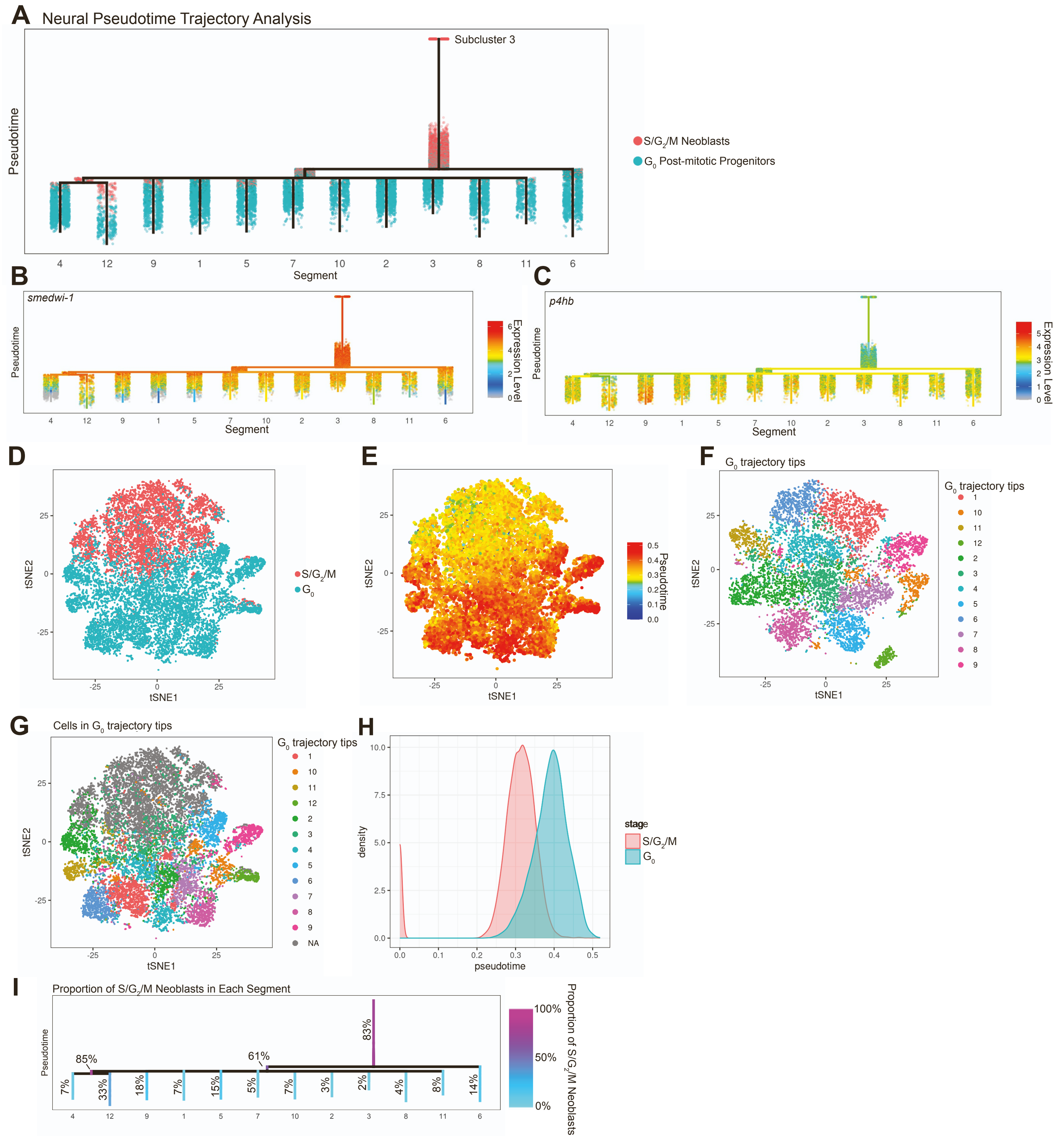

A
Neural Pseudotime Trajectory Analysis
Subcluster 3
Pseudotime
S/G2/M Neoblasts
G0 Post-mitotic Progenitors
4 12 9 1 5 7 10 2 3 8 11 6
Segment
B
smedwi-1
Pseudotime
Expression Level
Segment
C
p4hb
Pseudotime
Expression Level
Segment
D
tSNE2
tSNE1
S/G2/M
G0
E
tSNE2
tSNE1
Pseudotime
F
G0 trajectory tips
tSNE2
tSNE1
G0 trajectory tips
1 10 11 12 2 3 4 5 6 7 8 9
G
Cells in G0 trajectory tips
tSNE2
tSNE1
G0 trajectory tips
1 10 11 12 2 3 4 5 6 7 8 9 NA
H
density
pseudotime
stage
S/G2/M
G0
I
Proportion of S/G2/M Neoblasts in Each Segment
Pseudotime
85%
61%
83%
7% 33% 18% 7% 15% 5% 7% 3% 2% 4% 8% 14%
4 12 9 1 5 7 10 2 3 8 11 6
100%
50%
0%
Proportion of S/G2/M Neoblasts

Supplemental Figure 12, related to Figure 4. URD transcriptional pseudotime trajectory analysis for neurons

(A) Transcriptional pseudotime trajectory for neural-specified neoblasts and G_0 cells, rooted by neural neoblast subcluster 3.

(B) Expression of *smedwi-1* and (C) *p4hb* along pseudotime trajectories show gradients along pseudotime.

(D) tSNE dimensionality reduction of all muscle-specified cells included and their corresponding pseudotime (E).

(F) Louvain clustering of G_0 cells used as trajectory tips and their corresponding positions in tSNE space with specified neoblasts (G).

(H) Pseudotime distributions for $S/G_2/M$ neoblasts and G_0 post-mitotic progenitors.

(I) Percentages of $S/G_2/M$ neoblasts in each trajectory segment shows neoblasts present in all segments, including terminal segments with G_0 cells.

Figure S13

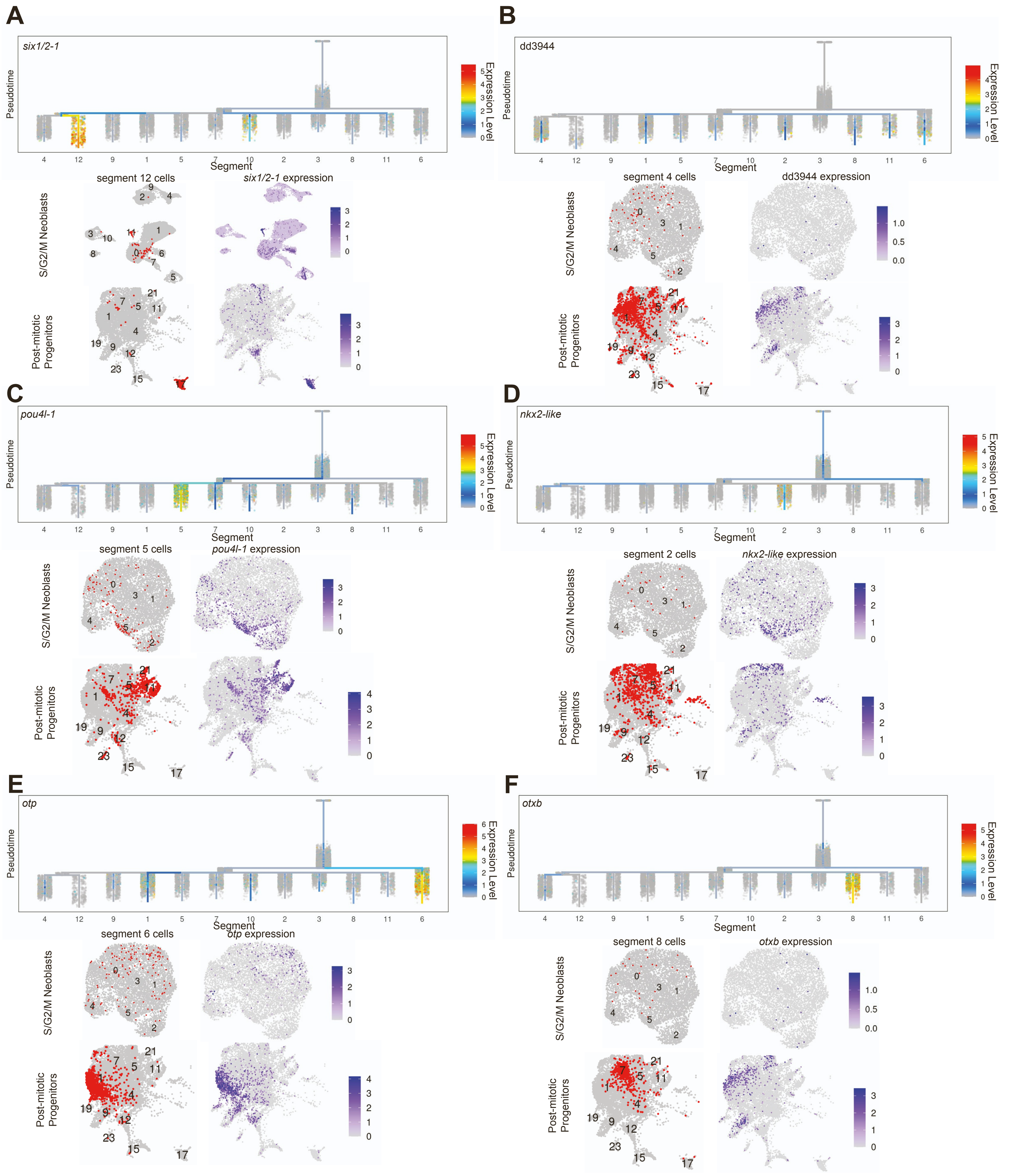

A
six1/2-1
Pseudotime
Segment
Expression Level
segment 12 cells
six1/2-1 expression
S/G2/M Neoblasts
Post-mitotic Progenitors
B
dd3944
segment 4 cells
dd3944 expression
C
pou4l-1
segment 5 cells
pou4l-1 expression
D
nkx2-like
segment 2 cells
nkx2-like expression
E
otp
segment 6 cells
otp expression
F
otxb
segment 8 cells
otxb expression

Supplemental Figure 13, related to Figure 4. Neural cell-type pseudotime trajectories from URD analysis

(A-F) Pseudotime trajectories of identified neural subtypes.

(A) Expression of *six1/2-1* is enriched in segment 12 (top), with cells in segment 12 existing in previously identified eye and sexual accessory cell- specified clusters (bottom-left), enriched in *six1/2-1* (bottom-right).

(B) Expression of dd_3944 is enriched in segment 4 (top), with cells in segment 4 existing in neural G_0 cluster 1, but not neoblasts (bottom-left), which is enriched in dd_3944 (bottom-right).

(C) Expression of *pou4l-1* is enriched in segment 5 (top), with cells in segment 5 existing separately in neural G_0 clusters 11, 21, 5, 4, and 12, as well as in a population of subcluster 5 neural neoblasts (bottom-left), which are enriched in *pou4l-1* (bottom-right).

(D) Expression of *nkx2-like* is enriched in segment 2 (top), with cells in segment 2 existing primarily in neural G_0 cluster 1, 7, and 5 (bottom-left), which are enriched in *nkx2-like* (bottom-right).

(E) Expression of *otp* is enriched in segment 6 (top), with cells in segment 6 existing in neural G_0 cluster 1, and not neoblasts (bottom-left), which is enriched in *otp* (bottom-right).

(F) Expression of *otxb* is enriched in segment 8 (top), with cells in segment 8 existing in neural G_0 cluster 7, and not neoblasts (bottom-left), which is enriched in *otxb* (bottom-right).

Figure S14

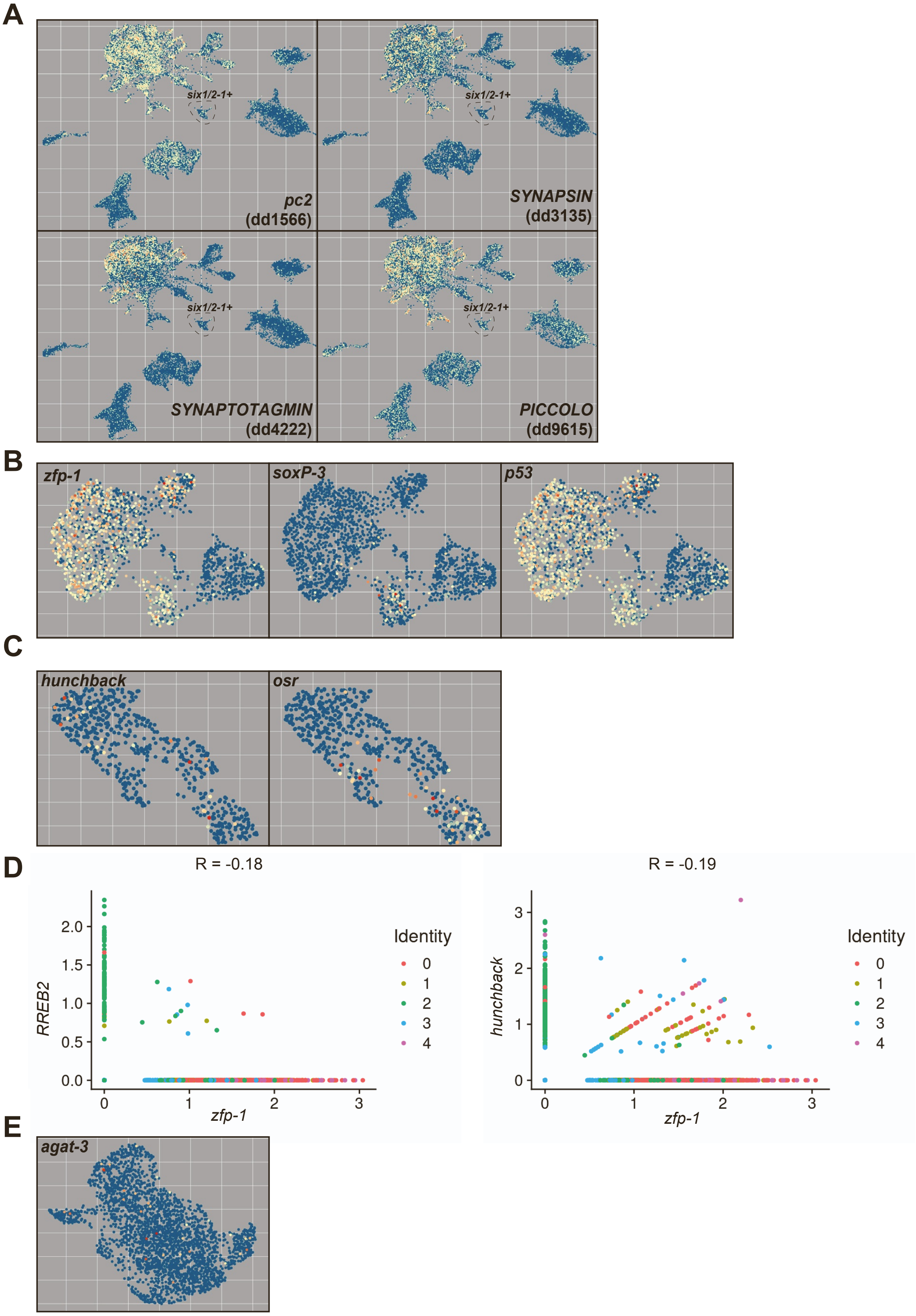
A
six1/2-1+
pc2
(dd1566)
SYNAPSIN
(dd3135)
SYNAPTOTAGMIN
(dd4222)
PICCOLO
(dd9615)
B
zfp-1
soxP-3
p53
C
hunchback
osr
D
R = -0.18
RREB2
zfp-1
Identity
0
1
2
3
4
R = -0.19
hunchback
zfp-1
E
agat-3

Supplemental Figure 14, related to Figures 5 and 6. Gene expression patterns and correlations in G_0 cells and subsets of $S/G_2/M$ cells.

(A) UMAP plot depicting expression of neural marker genes across all G_0 cells.

(B) UMAP plot depicting expression of *zfp-1*, *soxP-3,* and *p53* (canonical epidermal FSTFs) in intestinal neoblasts ($S/G_2/M$ cells).

(C) UMAP plot depicting expression of *hunchback* and *osr* in G_0 intestinal cells.

(D) Correlation values amongst $S/G_2/M$ cells for TFs associated with outer intestinal/basal cell fates (*RREB2, hunchback)* versus enterocyte states (*zfp-1*). TFs associated with cells of these two states are negatively correlated.

(E) Expression of *agat-3* in epidermal post-mitotic progenitors.
